# Characterization of the CAZy Repertoire from the Marine-Derived Fungus *Stemphylium lucomagnoense* in Relation to Saline Conditions

**DOI:** 10.3390/md18090461

**Published:** 2020-09-09

**Authors:** Wissal Ben Ali, David Navarro, Abhishek Kumar, Elodie Drula, Annick Turbé-Doan, Lydie Oliveira Correia, Stéphanie Baumberger, Emmanuel Bertrand, Craig B. Faulds, Bernard Henrissat, Giuliano Sciara, Tahar Mechichi, Eric Record

**Affiliations:** 1 Biodiversité et Biotechnologie Fongiques, Aix-Marseille Université, INRAE, UMR1163, 13288 Marseille, France; wissal.BEN-ALI@etu.univ-amu.fr (W.B.A.); david.navarro@inrae.fr (D.N.); elodie.drula@afmb.univ-mrs.fr (E.D.); annick.doan@inrae.fr (A.T.-D.); Emmanuel.Bertrand@Univ-Amu.Fr (E.B.); Craig.Faulds@Univ-Amu.Fr (C.B.F.); giuliano.sciara@inrae.fr (G.S.); 2 Laboratoire de Biochimie et de Génie Enzymatique des Lipases, Ecole Nationale d’Ingénieurs de Sfax, Université de Sfax, Sfax 3029, Tunisia; tahar.mechichi@enis.rnu.tn; 3 INRAE, Aix-Marseille Université, UMR1163, CIRM-CF, 13288 Marseille, France; 4 Institute of Bioinformatics, International Technology Park, Bangalore 560066, India; abhishek.abhishekkumar@gmail.com; 5 Manipal Academy of Higher Education, Manipal 576104, India; 6 Architecture et Fonction des Macromolécules Biologiques, Centre National de la Recherche Scientifique, Aix-Marseille Université, 13288 Marseille, France; Bernard.Henrissat@Afmb.Univ-Mrs.Fr; 7 USC AFMB, Institut National de Recherche Agronomique, 13288 Marseille, France; 8 AgroParisTech, Micalis Institute, PAPPSO, Université Paris-Saclay, INRAE, 78350 Jouy-en-Josas, France; lydie.oliveira-correia@inrae.fr; 9 Institut Jean-Pierre Bourgin, INRAE, AgroParisTech, CNRS, Université Paris-Saclay, 78000 Versailles, France; stephanie.baumberger@inrae.fr; 10 Department of Biological Sciences, King Abdulaziz University, Jeddah 21589, Saudi Arabia

**Keywords:** marine-derived fungus, *Stemphylium lucomagnoense*, saline adaptation, lignocellulose-degrading enzymes, secretome

## Abstract

Even if the ocean represents a large part of Earth’s surface, only a few studies describe marine-derived fungi compared to their terrestrial homologues. In this ecosystem, marine-derived fungi have had to adapt to the salinity and to the plant biomass composition. This articles studies the growth of five marine isolates and the tuning of lignocellulolytic activities under different conditions, including the salinity. A de novo transcriptome sequencing and assembly were used in combination with a proteomic approach to characterize the Carbohydrate Active Enzymes (CAZy) repertoire of one of these strains. Following these approaches, *Stemphylium lucomagnoense* was selected for its adapted growth on xylan in saline conditions, its high xylanase activity, and its improved laccase activities in seagrass-containing cultures with salt. De novo transcriptome sequencing and assembly indicated the presence of 51 putative lignocellulolytic enzymes. Its secretome composition was studied in detail when the fungus was grown on either a terrestrial or a marine substrate, under saline and non-saline conditions. Proteomic analysis of the four *S. lucomagnoense* secretomes revealed a minimal suite of extracellular enzymes for plant biomass degradation and highlighted potential enzyme targets to be further studied for their adaptation to salts and for potential biotechnological applications.

## 1. Introduction

Oceans, which represent approximately 70.8% of Earth’s surface, are not characterized by a high diversity of living organisms when compared with lands. Indeed, Grosberg et al. [1] reported that over the 1.5 million known species of macroscopic organisms on Earth, about 15% of species are found in oceans, 5% in freshwater, and a larger portion (approximately 80%) in terrestrial environments. Concerning the fungal kingdom, the same picture can be drawn, as about 1000 fungal species have been found in oceans and in freshwater, while the terrestrial habitats would account for more than 10^6^ different species [1]. A few studies have focused on the exploration of marine fungal diversity, and the number of described species is about a few hundred—Kohlmeyer et al. being one of the first pioneers in their isolation and identification [2]. Many fungi that are found in oceans are also found in terrestrial environments, e.g., *Fusarium*, *Aspergillus*, *Trichoderma*, or *Penicillium* [3]. This statement was illustrated by the work of Schoch et al. (2009), showing by a phylogenetic approach, including obligate marine species within the Dothideomycetes, that several have recently transitioned from terrestrial ancestors and that the corresponding transitions have occurred for multiple cases [4]. For this reason, for fungi isolated from aquatic environments, the term “marine-derived fungi” is often preferred and used in the literature, as their classification is not always well-established, although many efforts were made over the years [5]. From an ecological perspective, marine-derived fungi have been classified as obligate marine fungi, for those growing exclusively in a marine habitat, and facultative marine fungi, for those isolated from the freshwater or terrestrial origin, and also from the marine environment. [6,7]. Marine-derived fungi have been shown to be present in various habitats, such as decayed mangrove woods, algae, salt marshes, seagrasses, deep-sea sediments, sponges, and fishes [8]. Within these marine environments, fungal communities have adapted to diverse environmental conditions, very different from terrestrial ones, and notably high salinity, alkaline pH, low temperature, and eventually high pressure. For such a reason, their enzymes are expected to be of great interest for more profitable and ecological industrial bioprocesses, operated, for example, in non-pure waters at neutral or basic pH [9]. As such, marine fungi have been screened for retrieving original enzymes among amylases, chitinases, cellulases, xylanases, laccases, and many other potential biocatalysts [10]. In general, it was showed that their biochemical properties differ from those of catalysts isolated from corresponding strains isolated on lands, for instance, presenting activity in alkaline, low temperature, and saline conditions [10]. Marine-derived enzymes, as already assessed for their homologues from terrestrial fungi, could be of great interest in industrial sectors, such as the pulp and paper, tanning, and textile industries, whose processes involve high saline and alkaline conditions.

To survive under saline conditions, marine-derived fungi have likely developed physiological adaptations facilitating cellular homeostasis in these environments and secreted enzymatic machineries, active on biomass in marine habitats [11]. However, only a few studies describe the potential of marine-derived fungi to produce lignocellulolytic enzymes, which are key players of organic matter degradation in the sea [10,11,12]. A few of these strains have been studied at the genomic level, such as *Scopulariopsis brevicaulis* [13], *Cadophora malorum* [14] *Calcarisporium* sp., and *Pestalotiopsis* sp. [15]. For instance, it has been suggested that the marine-derived fungi *Pestalotiopsis* sp. and *S. brevicaulis* have conserved a metabolism relying on the breakdown of terrestrial plant biomass, while *Calcarisporium* sp. has adopted a different enzyme repertoire specialized into algal and animal biomass degradation. The latter reflects a lifestyle-oriented either towards parasitism or endophytic growth, or towards the necrotrophic use of algae or animals.

Few studies tried to gain insight into the adaptation of fungal enzymatic cocktails to degrade either terrestrial or marine complex carbon sources in saline conditions. Within the context of plant biomass biorefinery, wheat straw has already been used as a model terrestrial substrate to induce the production of fungal ligninolytic enzymes and their identification by a proteomic approach [16]. As a model for marine substrate, we selected is *Cymodocea nodosa*, an obligate underwater seagrass belonging to the family *Cymodoceaceae*, which plays essential roles in nutrient cycles, in preventing coastline erosion, and in providing habitats and hatcheries to marine life [17]. It naturally grows in Mediterranean coasts, as well as in the sites where we collected the five fungal isolates of our study. Finally, although micro-organisms drive the marine carbon cycle and subtle equilibria exist between microbial metabolism and carbon sequestration in coastal ecosystems [18], only a few examples can be found in the literature addressing the association of fungi to coastal plants [19], genomic analysis of marine-derived fungi [13,14,15], and proteomic studies of marine fungal enzymes involved in plant biomass degradation [12,20,21].

The main objectives of our work were to get insight into lignocellulose degradation by marine-derived fungi and to assess whether and how fungal enzymatic machineries adapt to saline conditions. In a previous study, five marine-derived fungal strains were isolated from Tunisian coasts and identified by a phylogenetic approach, and the adaptation of laccase-like activities in their secretome was assessed, in response to several factors, including salt conditions [22]. In this work, we study the growth of the five isolates and the tuning of lignocellulolytic activities under different conditions. *S. lucomagnoense* was then selected to follow the adaptation of its secretome composition when the fungus was grown on either a terrestrial (wheat straw) or a marine (seagrass) substrate, under saline and non-saline conditions.

## 2. Results

### 2.1. Effect of Carbon Sources and Sea Salt on Fungal Growth

**Figure 1:**
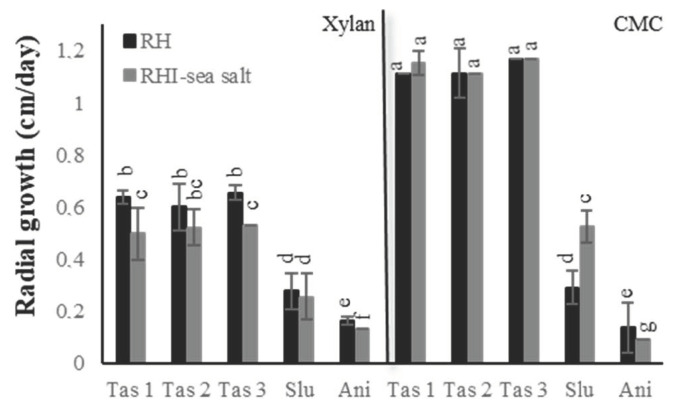
The radial growth rate of five isolated fungi: Three *Trichoderma asperellum strains* (*Tas*1, 2 and 3), *Stemphylium lucomagnoense* (*Slu*), and *Aspergillus nidulans* (*Ani*). Growth is measured on two different carbon sources, either xylan or carboxymethylcellulose (CMC), in the absence or presence of sea salt. Groups with different letters exhibit significantly different growth rates at a 95% confidence interval according to a Kruskhal–Wallis rank sum test, followed by a Dunn posthoc pairwise comparison test.

The radial growth of five fungal strains isolated from marine environments [22], namely, *Stemphylium lucomagnoense, Aspergillus nidulans,* and three *Trichoderma asperellum* strains (called *T. asperellum*1 (Tas 1), *T. asperellum*2 (Tas 2) and *T. asperellum*3 (Tas 3)), was evaluated. We followed and compared fungal growth on two different carbon sources, xylan, and carboxymethylcellulose (CMC), in the presence or absence of sea salt (Figure 1). The experiments were performed in triplicate for subsequent statistical analysis of data.

It can be seen that these strains were able to grow similarly on xylan in the absence and the presence of sea salt. However, while a slight reduction of fungal growth was observed for the three *T. asperellum* strains (from 0.63 cm/day to 0.49 cm/day) and *A. nidulans* (from 0.16 cm/day to 0.13 cm/day), *S. lucomagnoense* were not significantly affected by the salinity of the medium (0.27 cm/day). The five strains were also able to grow on CMC as the sole source of carbon, in the absence or presence of sea salt. Whereas for the three *T. asperellum* strains, no significant difference was observed in non-saline versus saline conditions, salt inhibited growth of *A. nidulans*, and improved that of *S. lucomagnoense*, from 0.29 cm/day to 0.52 cm/day.

### 2.2. Effect of Carbon Sources and Sea Salt on Lignocellulolytic Activities

**Figure 2:**
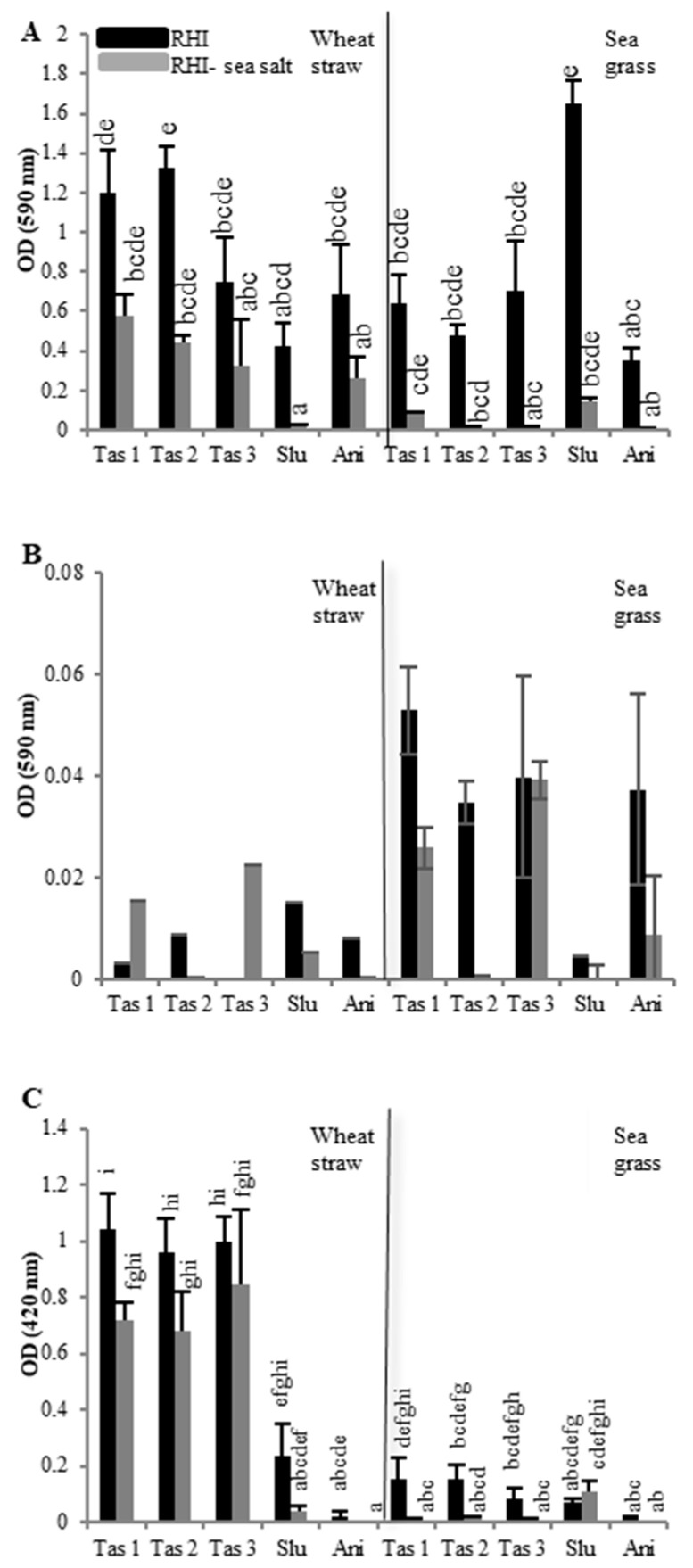
Screening of lignocellulolytic activities in the supernatant of liquid cultures of five isolated fungi (*Trichoderma asperellum*, *Tas*1, 2, and 3 *Stemphylium lucomagnoense*, *Slu* and *Aspergillus nidulans*, *Ani*). Enzymatic activity was tested after nine days of growth on either wheat straw or seagrass, in either non-saline (RHI, black bars) or saline (RHI-salt with 3% sea salt, grey bars). Xylanase, cellulase, and laccase-like activities were assessed spectrophotometrically, by following azo-xylan (**A**) and azo-CMC (**B**), hydrolysis at 590 nm, and ABTS oxidation (**C**) at 420 nm. For each activity, groups with different letters exhibit significantly different activity at a 95% confidence interval according to a Kruskhal–Wallis rank sum test, followed by a Dunn posthoc pairwise comparison test. The results of Dunn tests are to be found in the supplementary data (Table S1).

In parallel, lignocellulolytic activities were assessed for liquid cultures of the five strains grown in either non-saline (RHI medium) or saline (RHI plus 3% *w*/*v* sea salt) conditions on two different carbon sources, wheat straw, and seagrass. The potential towards the hydrolysis of azo-xylan (endo-xylanase activity) and azo-CMC (endo-cellulase activity), as well as towards the oxidation of 2,2′-azino-bis(3-éthylbenzothiazoline-6-sulphonique) (ABTS) (laccase-like activity), was assessed for secretomes from fungi grown for nine days on either wheat straw or seagrass (Figure 2). The experiments were performed in triplicate for subsequent statistical analysis of data.

Differences in xylanase activities (Figure 2A) were not pronounced when comparing cultures on wheat straw and seagrass, but saline conditions generally inhibited xylanase activities, as observed for laccase-like activities. When grown on wheat straw, *T. asperellum* strains showed the highest activities, followed by *A. nidulans* and *S. lucomagnoense*, respectively. On the other hand, azo-xylan hydrolysis from seagrass cultures of *S. lucomagnoense* was by far the most efficient. For cellulase activities, no significant differences could be seen, probably because the standard deviations were high in our test conditions. It would appear that cellulase activities were higher in cultures grown on seagrass in non-saline conditions (Figure 2B), and that *T. asperellum* and *A. nidulans*, showed the highest ones For laccase-like activities (Figure 2C), results show that globally in saline condition, the culture medium reduced the secretome activities for all strains except for *S. lucomagnoense* cultured on seagrass, confirming our previous results [22]. In general, the three *T. asperellum* strains showed the highest activities, and *A. nidulans* the lowest. In addition, laccase-like activities were much higher for cultures grown on wheat straw.

### 2.3. cDNA Library Construction and Sequencing, and Annotation of the Lignocellulolytic Enzyme Set from the Stemphylium Lucomagnoense Proteomes

A more detailed analysis of secreted enzymes in response to growth conditions was conducted on one strain only. In our search of a secreted enzymatic machinery with original features, with a focus on enzymes with novel properties, *S. lucomagnoense* was selected based on: (i) Its growth response in saline conditions that was either unaffected or improved on xylan and CMC, respectively (Figure 1); (ii) its highest xylanase activity among the five strains from cultures on seagrass; both in the presence and absence of sea salt (Figure 2A); and (iii) its secreted laccase-like activity which was found to be slightly improved in cultures on seagrass with sea salt (Figure 2C). These characteristics, in fact, point to the potential retrieval of novel microbial and enzymatic features of interest for industrial applications, deriving from *S. lucomagnoense* adaptation to the marine environment. As no genomic data are available for this fungus, a representative cDNA library was constructed and sequenced using the approach developed for the mangrove fungus, *Pestalotiopsis* sp. [12]. Briefly, the transcriptome (total RNAs were extracted from the various growth condition sand pooled together before sequencing) was sequenced to prepare the cDNA library (replacing the genome) to identify the proteins obtained from the proteome and not to quantify the expression of the various genes. In addition, the cDNA library was normalized so that the read counts of each transcript could not be determined. The datasets of this study were deposited under National Center for Biotechnology Information (NCBI) BioSample accession ID: SAMN15897915 with corresponding NCBI BioProject accession ID: PRJNA659110. The corresponding proteins were identified using the CAZy database (www.cazy.org), and the resulting protein database was used to identify, by mass-matching, the proteins obtained from a proteomic mass spectrometry analysis.

To capture the largest possible number of transcripts, a normalized cDNA library was prepared from total RNAs isolated from pooled samples taken after 2, 4, 6, 8, and 10 days of culture for each condition. This library was sequenced using the Illumina technology (2 × 150 bp) (Illumina, San Diego, CA, USA) with an average of 300 million paired-end sequences. After the initial quality assessments, de novo transcriptomic assembly was achieved using the de novo assembler suite from the CLCBio Genomic Workbench version 11 (https://digitalinsights.qiagen.com) (Qiagen, Hilden, Allemagne). In this de novo transcriptomic assembly, a total of 520,000,000 reads were assembled in 350,657 contigs. The N50 for the contigs is 504 bp, and an average length of 316 bp (Table 1). The assembled contigs sequences are available at GitHub URL as https://github.com/drabhishekkumar/Stemphylium-lucomagnoense-transcriptomics.

In order to obtain an expert and accurate annotation of the lignocellulolytic enzymes present in the transcriptome, translated transcripts were compared to the entries in the CAZy database (www.cazy.org). Using 1D electrophoresis followed by LC-MS/MS analysis, 51 secreted *S. lucomagnoense* proteins were identified as belonging to one of the CAZy classes. CAZymes, including glycoside hydrolases (GHs), carbohydrate esterases (Ces), polysaccharide lyases (PLs), carbohydrate-binding modules (CBMs), glycosyl transferases (GTs), and auxiliary activities (Aas), are, in fact, microbial enzymes involved in complex carbohydrate breakdown and assembly, and in particular in lignocellulose degradation and transformation. Among the *S. lucomagnoense* CAZymes, 38 GHs, 3 Ces, 1 PLs, and 7 Aas and 13 CBMs, appended to an enzymatic module or not, were identified, including multi-modular proteins (Table 2).

### 2.4. Enzyme Distribution in Secretomes from Cultures on Wheat Straw and Seagrass, in Saline and Non-Saline Conditions

**Figure 3:**
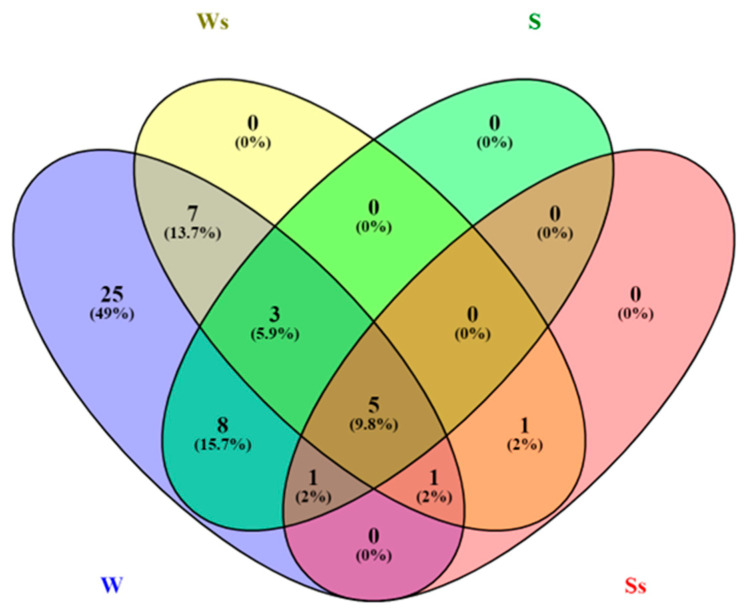
Venn diagram representing numbers of CAZymes identified in *Stemphylium lucomagnoense* secretomes grown on either seagrass (S/Ss) or wheat straw (W/Ws), in saline (Ss/Ws) and non-saline (S/W) conditions.

To gain insight into the *S. lucomagnoense* enzyme machinery acting on lignocellulose, the distribution of enzymes retrieved by proteomic analysis in the four different growth conditions was determined. This analysis was carried out on four specific secretomes, i.e., *S. lucomagnoense* cultures produced on either wheat straw (as model terrestrial plant biomass substrate) or seagrass (as model marine substrate), with or without 3% sea salt (Figure 3). Out of the 51 identified proteins, 50 (98%) and 17 (33%) proteins were produced on wheat straw in non-saline and saline conditions, respectively, showing a negative pressure of sea salt on protein production (Figure 3). For seagrass cultures, overall, even fewer proteins were identified in the secretomes produced in non-saline and saline conditions, i.e., 17 (33%) and 8 (15.6%), respectively. Five CAZymes were produced in all the tested conditions (10%).

In more detail, all the identified proteins were produced in cultures grown on wheat straw, and only 19 (37%) on seagrass (Figure 3). No protein was exclusively found from cultures grown on seagrass, while 25 (49%) were exclusively found in wheat straw cultures, for instance, one member of family GH31 (APMZ2_prot29015) and three of family GH3 (APMZ2_prot3532, 3750, 15280) for the most abundant proteins (Table 2). For proteins identified in secretomes grown on wheat straw, 34 (66.7%) were exclusively identified in non-saline cultures, as for instance, one member of the family GH78 (APMZ2_prot5721). For seagrass cultures, only two proteins were exclusively identified in saline conditions. Only one protein, PL7_4 (APMZ2_prot21161), was exclusively identified in saline conditions from cultures grown on both wheat straw and seagrass.

**Figure 4:**
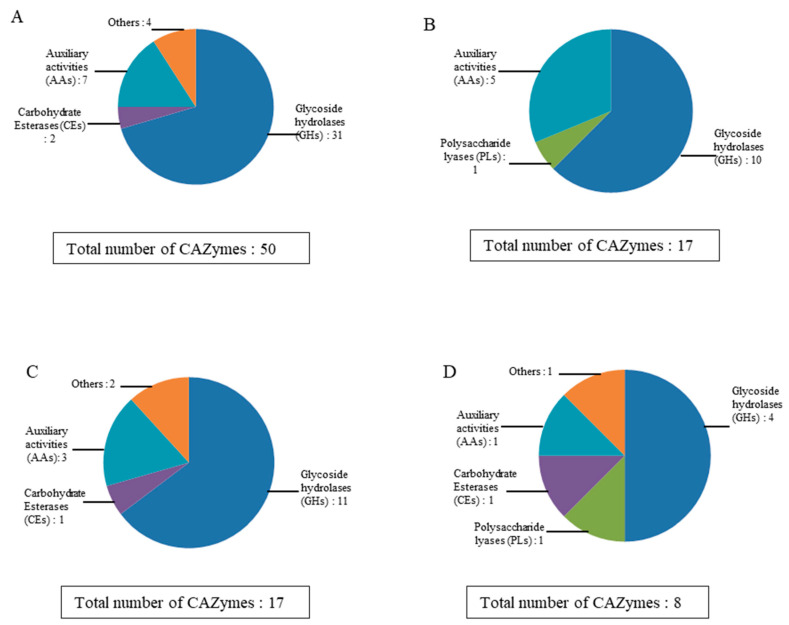
Distribution of CAZymes in *Stemphylium lucomagnoense* secretomes grown on either wheat straw in non-saline (**A**) and saline (**B**) conditions, or on seagrass in non-saline (**C**) and saline (**D**) conditions. “Others” include enzymatic activates and proteins, such as PLs and CBMs.

Considering the CAZyme family repartitions in cultures grown on wheat straw, the production of GHs was drastically inhibited with 37 to 11 members, and a lower pressure was observed on Aas (7 to 5) (Figure 4). Of the two CE (carbohydrate esterase) members found in wheat straw cultures, none were found in saline conditions. Related to seagrass cultures, the ratio was equivalent to 11 GH (glycoside hydrolase) members against 4 found in non-saline versus saline conditions. In addition, one-third of the Aas was found in saline conditions. The unique CE member identified in seagrass cultures was found in both conditions. The only CAZy member found exclusively in saline conditions is the PL member already cited (APMZ2_prot21161).

Cellulases are a wide and heterogeneous group of enzymes, encompassing a large number of phylogenetically diverse proteins families. In *S. lucomagnoense* secretomes, only GH1 (APMZ2_prot27520) and GH3 (APMZ2_prot1520, 3532, 3750, 15280) accessory enzymes to cellulose degradation were found, but no members of families GH5, 6, 7 and 12 for instance, unlike normally found in most terrestrial lignocellulose degrading fungi. The five GH1 and GH3 enzymes were mainly produced when *S. lucomagnoense* was grown on wheat straw, with the exception of protein 1520, which was also found when seagrass was used as a growth substrate. No cellulases were identified in saline conditions though. Besides cellulases, other carbohydrate degrading enzymes were present in the secretome of *S. lucomagnoense*, such as GH18 members. Family GH18, generally found in filamentous fungi, includes chitinases and can be appended to carbohydrate-binding modules (CBMs) of family CBM18. Modules of family CBM18 have been demonstrated to bind to chitin and to be involved in fungal processes, such as cell wall degradation and modification, spore germination, tip growth, hyphae branching, spore differentiation, and autolysis [23]. In the *S. lucomagnoense* proteome, the CBM18 module was found in two multi-modular proteins (APMZ2_prot275 and 7701), fused to carbohydrate esterase domains (CE4). Family CE4 contains putative chitin deacetylases, which are known to be involved in cell-wall chitosan biosynthesis in Mucorales, by action in tandem with chitin synthases of the glycoside transferase family GT2 [24]. Strikingly, another carbohydrate-binding module, CBM50, was found as a unique module or in tandem as a trimodular protein (APMZ2_prot23159 and 18739, respectively). CBM50 is a module of approximately 50 residues, generally grafted to various enzymes that cleave chitin or peptidoglycan, including families GH18, 19, 23–25, and 73. When not associated with other enzymatic modules, CBM50, like CBM18, is involved in the recognition or binding of chitin and prevents hydrolysis of the fungal cell wall by plant chitinases, therefore interfering with chitin-triggered host immunity [25].

When considering candidate hemicellulases, two putative xylanases were identified as members of family GH10 (APMZ2_prot14594 and 411), appended to a carbohydrate-binding module of family CBM1, and one α-l-arabinofuranosidase (family GH51, APMZ2_prot9402). Family GH43 was also particularly well represented in the *S. lucomagnoense* secretomes, with four representatives. This family includes α-l-arabinofuranosidase, β-d-xylosidase, α-l-arabinanase, β-d-galactosidase, and many other enzymes involved in hemicellulose and pectin debranching and degradation [26]. Among the four GH43 representatives, the most abundant was APMZ2_prot17304 (GH43_24-CBM35), which is a hypothetical β-1,3-galactosidase fused to a CBM35 module, known to bind 1,3-β-d-galactose. The second most abundant representative of the family in *S. lucomagnoense* belongs to subfamily GH43_26 (APMZ2_prot21603), and the two-last retrieved GH43 domains were associated with a bimodular protein, GH43_22-GH43_34 (APMZ2_prot23037). GH43_34 modules are most associated with another GH43 domain, but the resulting fusion proteins are not characterized, although bacterial GH43_34 members have been described as α-l-arabinofuranosidases [27]. All *S. lucomagnoense* enzymes involved in hemicellulose degradation are also found mainly in cultures grown on wheat straw, with the exception of two proteins (APMZ2_prot14594 and 17304, i.e., the most abundant hemicellulases in the analyzed secretomes), which were found both on seagrass and wheat straw.

Among Auxiliary Activities (Aas), a copper radical oxidase (CRO, APMZ2_prot912) was produced in a relatively large amount as compared to other representatives in all the culture conditions tested. This protein is predicted to be phylogenetically related to glyoxal oxidases (GLOXs, CAZy subfamily AA5_1) [28]. CROs are metalloenzymes containing one copper metal ion. While GLOX enzymes have been biochemically characterized [29,30], other copper radical oxidases (CRO1 to CRO6) are still uncharacterized proteins of unknown function [31]. Two more proteins well expressed from *S. lucomagnoense* (APMZ2_prot27712 and 24323) were identified as putative aryl alcohol oxidases (AAOs) (CAZy subfamily AA3_2). These two proteins respectively appeared in cultures grown wheat straw either with or without sea salt, or on both wheat straw and seagrass. The AA3 family is still not completely characterized, and recently 3 AAO-like enzymes were shown to act as aryl alcohol quinone oxidoreductases (AAQOs), displaying a preference for quinones as electron acceptors, and lower reactivity towards molecular oxygen as typically observed for AAO [32]. In addition, glucose oxidases and dehydrogenases were found in subfamily AA3_2, and as such, further biochemical characterization will be required to precisely annotate these two *S. lucomagnoense* enzymes. A hypothetical pyrroloquinoline quinone (PQQ)-dependent oxidoreductase (AA12, APMZ2_prot4629) was also identified in *S. lucomagnoense* secretomes. PQQ-dependent enzymes were only recently found and described in fungi. They catalyzed the oxidation of various sugars, and their three-dimensional structure was recently elucidated [33,34]. One member of this family, the PQQ-dependent pyranose dehydrogenase from *Coprinopsis cinerea* (*Cc*PDH), was demonstrated to sustain the catalytic cycle of a lytic polysaccharide mono-oxygenase (LMPO) [35]. In fact, LPMOs can complete their catalytic cycle and catalyze the oxidative cleavage of glycosidic bonds only if external electron donors are available [36,37]. As an example, it was shown that *Cc*PDH activates the C-1-oxidizing LPMO 9F and the C-4-oxidizing LPMO 9C from *Neurospora crassa* by direct electron transfer from the AA12 and through the AA8 (c cytochrome) domain [35]. In our study, the AA12 protein was found in all conditions tested, and one LMPO (CAZy family AA11, APMZ2_prot21080) was also identified in secretomes produced on wheat straw in either saline or non-saline conditions. One AA11 LMPO was demonstrated to be involved in the hydrolysis of chitin chains, and C-1 oxidative cleavage has been demonstrated for a member of this family from *Aspergillus oryzae* [38]. Chitin is present in the exoskeleton of arthropods, as well as in the fungal cell wall. The function and the enzymatic partners of AA11 enzymes in marine fungi are still to be elucidated. However, AA3_2 and AA12 enzymes found in *S. lucomagnoense* secretomes are reasonably good candidates [37].

Among the most abundant proteins, a member of family GH55 (APMZ2_prot 15973) was identified. Members of this family include endo/exo-β-1,3-glucanases, which have a general role in morphogenesis, and more specifically, in cell wall softening during conidial formation or fruiting body development [39,40]. They were also shown to possess potential antifungal activity with a degradative effect on cell walls [41]. GH55 enzymes display a broad range of specific activities, including laminarin hydrolase activities. Laminarin is a storage glucan found in brown algae (*Phaeophyceae*) and is formed of a glucan chain, made of β-1,3-linked glucose, with β-1,6 branches. It represents 10–45% of microalgal biomass, and as such, a key component of food chains and the carbon cycle in the oceans [42]. Not surprisingly, these enzymatic activities have already been found in marine-derived fungi [43], as well as amylase activities [44]. These activities are also present in *S. lucomagnoense* secretome, with a GH15-CBM20 glucoamylase (carrying a starch binding module, APMZ2_prot13181) and a GH13_1 β-amylase (APMZ2_prot2472). To conclude, the less expressed *S. lucomagnoense* protein is a member of family PL7 (APMZ2_prot21161) identified in cultures grown on both wheat straw and seagrass, but exclusively in saline conditions. This enzyme is a putative alginate lyase, an important enzyme used by marine-derived fungi to degrade brown algae biomass [20].

## 3. Discussion

The objective of the present study was to provide new insight into the lignocellulose enzyme machinery of marine-derived fungi, to understand how they adapted to process biomass in saline conditions. For this purpose, we screened five fungal strains isolated from the Tunisian coast [22]. Based on a microbial approach and on enzyme activity screening, we selected *S. lucomagnoense* for its adapted growth on xylan in saline conditions, its higher xylanase activity, and its improved laccase (seagrass-containing cultures) and cellulase (wheat straw-containing cultures) activities in the presence of sea salt. The screening of marine-derived isolates showed that the five strains can use xylan and cellulose for growth, and that they secreted the corresponding activities in the extracellular medium to degrade and use these substrates. Other marine-derived fungi, such as *Cadophora* sp. TS 2, *Emericellopsis* sp. TS 11 and *Pseudogymnoascus* sp. TS 12 isolated from the deep-sea sponge *Stelletta normani*, were previously demonstrated to possess lignocellulolytic properties without striking differences in their growth profiles, on either CMC or xylan [11]. This study showed no effect on fungal growth for sodium chloride up to 0.5 M, but growth inhibition occurred for NaCl concentrations above 1 M. Wang et al. (2016) [45] also studied the effect of various substrates on the growth of six marine-derived fungi: *Calcarisporium* sp. KF525, *Tritirachium* sp. LF562, *Bartalinia robillardoides* LF550, *Penicillium pinophilum* LF458, *Scopulariopsis brevicaulis* LF580 and *Pestalotiopsis* sp. KF079. For almost all fungal strains, grown in 3% sea salt, a preference for xylan over cellulose was generally showed, unlike in our results. Batista-Garcia (2017) [11] also showed no impact on cellulase and xylanase activities at 0.5 M NaCl. At higher salt concentrations, however, the two enzyme profiles diverged, showing strong inhibition of cellulases, and different behavior of xylanases: Strong inhibition of xylanases from *Cadophora* sp. TS, slight activation of those from *Pseudogymnoascus* sp. TS 12, and activation between 1 and 2 M NaCl of xylanases from *Emericellopsis* sp. TS 11, suggesting that each strain adopted a different enzymatic strategy for degrading plant biomass in saline conditions. Arfi et al. [12] reported that sea salt (3% *w*/*v*) has a negative effect on cellulase and xylanase activities of the mangrove fungus *Pestalotiopsis* sp. NCi6 without effect on fungal growth rate. Even with 6% salt, a 2-day-latency phase was observed—but without impact on the final growth rate. Arfi et al. [12] suggested that this fungal ability to metabolize the carbohydrate substrates was not affected by salt. Finally, other studies of xylanase production or of xylanase activities of cell-free supernatants could be found from other marine-derived fungi as from *Trichoderma pleuroticola* 08ÇK001 strain isolated from Mediterranean coastal sediments [46], but a few reports are available for cellulases [47]. Several reports previously focused on laccase-like activities, summarized in a recent review on marine-derived micro-organisms [48], and one specifically dedicated to marine-derived fungi [10]. For instance, Bonugli-Santos et al. reported laccase activities from *Marasmus* sp., *Peniophora* sp. And *Tinctoporellus* [49], but they pursued no further characterization. They also optimized the production of laccases from *Mucor racemosus* with an optimum at 4 M NaCl [50]. In contrast, a laccase from *Trematosphaeria mangrovei*, isolated from mangroves, was inhibited (50%) by 1 mM NaCl only [51]. Finally, saline conditions enhanced the activities of two laccases, the purified forms of the marine-derived fungus *Pestalotiopsis* sp., for sea salt concentration up to 5% [52].

Assaying enzyme activities in fungal secretomes to study the fungal adaptation to saline environments has limitations, as the method reflects the global activity of potentially diverse enzymatic machineries possessing different properties. To better understand the adaptation of *S. lucomagnoense* at the enzyme level, we used proteomic analysis, as previously done for the mangrove fungus *Pestalotiopsis* sp. NCi6 [12]. This mangrove fungus belongs to the order *Pleosporales* [17,53] (Dothideomycetes), which includes marine-derived fungi and a majority of terrestrial species [4]. We first sequenced and assembled the transcriptome of *Pestalotiopsis* sp. Transcribed genes were identified and functionally annotated using the CAZy database [54] and used to identify secreted enzymes by matching LC-MS/MS data.

The correlation between the CAZyme repertoire identified in secretomes and growth phenotypes is not always clear, and the absence or incomplete transcriptomic and proteomic data might be claimed responsible in these specific cases, yet comparing proteomes from the four culture conditions provides an interesting insight into the adaptation on fungal secretomes to saline conditions. As an example, higher xylanase activities were found in secretomes from cultures in non-saline conditions, on both wheat straw and seagrass, indicating inhibition by sea salt added to the culture medium. Accordingly, APMZ2_prot14594 and prot411 (family GH10) are identified in proteomic data from these conditions (the latter on wheat straw only). A similar scenario was found for three GH43 xylanases. Intriguingly, xylanase activities were higher in cultures grown on seagrass, but the number of spectra was lower (for APMZ2_prot14594 and prot17304), suggesting lower protein secretion, but higher enzyme activity in saline conditions. To check this hypothesis, gene cloning and recombinant protein purification would be required, followed by enzyme characterization to understand the effect of salt on enzymatic activity. Using this approach, in a previous study on two laccases from *Pestalotiopsis* sp. KF079, isolated from the Baltic sea mudflats, we observed concomitant enzyme activation in saline conditions and the presence of highly charged, negative electrostatic protein surfaces [52], as studied at the molecular level for a cellobiohydrolase (GH7) from the marine wood borer *Limnoria quadripunctata* [55]. Other hemicellulases were identified, such as the feruloyl esterase (APMZ2_prot7619 produced in both carbon sources without salt) as the two other hemicellulases candidates (APMZ2_prot17211 and APMZ2_prot29026) that were identified only on wheat straw cultures without salt.

Cellulase activities of *S. lucomagnoense* were rather low compared to other marine-derived isolates screened in our study. Only one and four members of families GH1 and GH3, enzymes accessory to cellulose degradation, respectively, were identified in secretomes. No representatives of GH families 5, 6, 7, and 12 were found, unlike usually seen with terrestrial fungi. Four members were only found in cultures without salt of *S. lucomagnoense* grown on wheat straw, and one (APMZ2_prot1520) from both substrates, meaning that the cellulolytic ability of the fungus is limited or that these families contain cellulases never described before. Considering the second hypothesis, the cloning of the corresponding genes and the characterization of the recombinant enzymes will be required. In line with these data, cellulase activity was mainly detected in cultures without sea salt on wheat straw, and less, on seagrass only. Although a similarly low cellulase activity was found in cultures on wheat straw in saline conditions, no candidate cellulase was identified in the secretome. This possibly points to the sensitivity limit of our combined transcriptomic-proteomic approach, because of undetected transcript domains, as previously hypothesized in our previous study [12].

Although laccase-like activity was detected in *S. lucomagnoense* secretomes from cultures grown on either wheat straw or seagrass, in any secretome, neither laccase-like (AA1) nor peroxidase-like (AA2) enzymes, also able to oxidize ABTS, were identified. This finding suggests that the oxidase responsible for ABTS oxidation was undetected, maybe for the reason just cited above as several transcripts were identified as encoding putative laccases in *S. lucomagnoense* transcriptome. Analyzing fungal secretion of Auxiliary Activities, the number of candidates was higher in wheat-containing cultures, but a large part was also found in seagrass-containing cultures. The number of identified proteins was reduced in saline conditions, but still, more than 50% of them were found, suggesting that *S. lucomagnoense* can cope with marine mimicking conditions. Something similar was also seen in the work of Arfi et al. [12] on the mangrove fungus *Pestalotiopsis sp*. In that work, we only tested the effect of saline conditions on fungal physiology, while in our study, we combined the salt and the carbon substrate effects. Two enzymes, APMZ2_prot912 (family 5_1, CRO) and APMZ2_prot4629 (family AA12, pyrroloquinoline-quinone (PQQ)-dependant oxidoreductase), were found in all conditions tested at pretty high yields. Recently, it was shown that a (PQQ)-dependent pyranose dehydrogenase (family AA12) from *Coprinopsis cinerea* was able to activate a family LPMO from *Neurospora crassa* [35]. One candidate LPMO (APMZ2_prot21080, family AA11), was also identified in *S. lucomagnoense* cultures grown on wheat straw, both in the presence and absence of sea salt. Although further investigation will be necessary to demonstrate the potential synergy between the two *S. lucomagnoense* enzymes, like for terrestrial fungi, fungal cellulose degradation seems to rely on LMPOs, which are copper oxidases classified among the AA section of the CAZy database. In a previous study, we demonstrated that an LPMO was produced from *Pestalotiopsis* sp. NCi6 was able to cleave polymeric cellulose in the presence of up to 6% sea salt [56]. Salt resistant enzymes are interesting potential biocatalysts as they might achieve saccharification and other industrial processes in seawater, with improved ecological footprints.

Focusing on family AA3_2 (aryl alcohol and glucose oxidases and dehydrogenases), we can notice different enzymatic profiles in different growth conditions: APMZ2_prot27112 is only produced on wheat straw, with or without salt; APMZ2_prot24323 is secreted on seagrass without salt, as well as on wheat straw with salt; APMZ2_prot2229 only on wheat straw without salt. This diversity in the enzymatic response of *S. lucomagnoense* within a specific enzyme family illustrates a putative adaptation to the growth conditions: Nature of the carbon source and physico-chemical conditions, such as salt. More detailed studies will be necessary for in-depth understanding of this adaptation at the enzyme level.

The four most secreted proteins (APMZ2_prot13181, 912, 15973, 2472) were produced in all the conditions investigated. They correspond to GH55 (β-1,3-glucanase), probably involved in fungal morphology, GH15-CBM20 (glucoamylase fused to a starch binding module), and GH13_1 (β-amylase). Similar enzymatic activities were already found to be highly expressed in other secretomes, such as described for the coprophilous ascomycete *Podospora anserina,* cultivated on wheat straw, Avicel, sugar beet pulp, and for the corn pathogen *Fusarium verticillioides* [57,58]. These enzymatic activities were instead absent in secretomes from the white-rot basidiomycete *Phanerochaete chrysosporium* [59]. Although further experiments will be needed to understand their role in *S. lucomagnoense* physiology, these constitutively secreted proteins might play a central metabolic role. One protein only was found for both substrates exclusively in saline conditions: APMZ2_prot21161, the less abundant enzyme revealed by our proteomic analysis, a putative alginate lyase (CAZy subfamily PL7_4). Such an enzymatic activity was already demonstrated to be key for the marine-derived fungus *Paradendryphiella salina* to degrade several types of brown algae polysaccharides [20], despite a minimal CAZyme repertoire, which is a typical feature of saprobic and plant pathogenic ascomycetes. In a previous work, we also found four putative PL7_4 genes in the genome of a marine-derived fungus, *Calcarisporium* sp. KF525, also pointing to a possible capacity to degrade algal biomass [15]. For comparison purposes, the marine-derived fungus *Pestalotiopsis* sp. KF079, isolated in the same environment [15], and *Scopulariopsis brevicaulis* [13] adopted instead a different and larger CAZy repertoire, enabling the breakdown of terrestrial plant biomass.

In conclusion, our work highlights the potential of proteomic analysis of marine-derived fungi to gain insight into microbial metabolism and adaptation to the marine environment. To achieve this goal, further characterization of several novel enzymes identified in *S. lucomagnoense* secretomes will be necessary, i.e., cloning and overexpressing the related genes in appropriate hosts, characterizing the recombinant enzymes from the kinetics to the 3D-structure, and elucidating the molecular determinants responsible for the properties of these enzymes. Finally, the results of our study contribute to more distant fields of research, such as the study of the carbon cycle and carbon sequestration, the understanding of microbial communities, and the discovery of novel biocatalysts for industrial applications.

## 4. Materials and Methods

### 4.1. Radial Growth Rate Determination

The specific radial growth rate of five isolated fungal strains (*T. asperellum*1, *T. asperellum*2, *T. asperellum*3, *S. lucomagnoense,* and *A. nidulans*) was determined on Petri dishes, by inoculating 6 mm plugs obtained from one-month-old fungal pre-cultures grown in Vogel’s medium [60] into new plates containing the same medium supplemented with either 2% (*w*/*v*) carboxymethycellulose (CMC) (Sigma-Aldrich, Saint-Louis, MO, USA) or 2% (*w*/*v*) xylan (Sigma-Aldrich, Saint-Louis, MO, USA) as sole extra carbon sources. The effect on the growth of saline conditions was determined by adding 3% (*w*/*v*) sea salt to the growth medium of half of the samples. The growth rate at 30 °C was expressed as cm/day. The diameter of the colony around the inoculation plug was measured every 24 h for 10 days. Experiments were performed in triplicate for subsequent statistical analysis of data.

### 4.2. Screening of Lignocellulolytic Activities on Wheat Straw and Seagrass

The culture supernatant of five strains grown on RHI medium (containing per liter: 18 g Bacto Agar, 1 g malt extract, 1 g yeast extract, and 40 g substrate) [12] at pH 5.5 was used to follow the presence of lignocellulolytic enzymes in fungal secretomes grown on two different plant biomasses. Wheat straw was used as the terrestrial reference substrate, because it is an important agro-industrial residue and because it was previously demonstrated to be a good inducer of lignocellulolytic enzymes [57]. Seagrass (*Cymodocea nodosa*) was the chosen marine substrate, as it was found in the same area where the marine strains were collected. The two plant substrates were washed with distilled water and then kept for 24 h in a 70 °C oven for drying, then grinded using an analytical mill (IKA A11 basic, Sigma-Aldricht, Saint-Louis, MO, USA) and the resulting fragments were then sieved with a metal mesh for a size between 0.2 and 0.8 mm. They were finally sterilized twice for 1 h at 110 °C. Each strain was inoculated in three 250 mL baffled Erlenmeyers (three biological replicates) containing 50 mL of medium and incubated at 30 °C in the dark under agitation at 50 rpm (Infors, Massy, France). The inoculum for each Erlenmeyer consisted of two agar plugs (6 mm diameter), cut from the growing edge of a plate stock culture, homogenized in 1 mL of water in Lysing Matrix A tubes with a FastPrep Instrument (MP Biomedicals, Illkirch-Graffenstaden, France) at 4 m s^−1^ for 15 s. Depending on the experiment, the ‘RHI’ medium was supplemented with 3% (*w*/*v*) of sea salt (Sigma-Aldrich, Saint-Louis, MO, USA).

Enzymatic activities were assessed by collecting 1 mL of each culture supernatant every 2 days over 12 days. After centrifugation at 10,000× *g* for 5 min, the supernatant was collected and used in several enzymatic assays (two technical replicates). For each culture condition, the measurement was performed in triplicates (biological replicates). The laccase-like activity was tested by monitoring the oxidation of 2,2′-azino-bis (3-ethylbenzothiazoline-6-sulphonic acid) (ABTS; Sigma-Aldrich, Saint-Louis, MO, USA). Twenty microliters of culture supernatant were added to 130 µL of 0.1% ABTS in 100 mM citrate-phosphate buffer pH 5, and optical density was measured at 420 nm after 30 min of incubation at 30 °C. Endo-1,4-β-d-glucanase (endo-cellulase) (Megazyme, Libios, Vindry-sur-Turdine, France) and endo-1,4-β-d-xylanase (endo-xylanase) (Megazyme, Libios, Vindry-sur-Turdine, France) activities were assessed by measuring, respectively, the hydrolysis of 1% (*w*/*v*) azo-CMC (Megazyme, Libios, Vindry-sur-Turdine, France) and 1% (*w*/*v*) azo-xylan (birchwood, Megazyme, Libios, Vindry-sur-Turdine, France) in water. Twenty microliters of culture supernatant were added to 130 µL of each azo-substrate solution and incubated 30 min at 30 °C. 375 µL of 95% (*v*/*v*) ethanol was then added, in order to stop the enzymatic reaction by protein denaturation/precipitation, and after centrifugation at 5000× *g* for 10 min, the corresponding supernatant was collected and optical density measured at 590 nm.

### 4.3. Statistical Analysis of Data

The statistical analysis of data on radial growth rate was carried out using the linear regression model in R to compute the radial growth rate and its associated 95% confidence interval. The statistical analysis on cellulase, xylanase, and laccase-like activities were carried out with non- parametric Kruskal–Wallis test [61] (*p* < 0.05), followed by post hoc Dunn pairwise comparison test [62] when differences were previously found significant using R version 3.6.3 and Rstudio version 1.2.5.033.

### 4.4. RNA Extraction, cDNA Library Construction, Sequencing, Assembling and Annotation

*S. lucomagnoense* was cultivated in 50 mL of liquid medium RHI, with or without 3% (*w*/*v*) sea salt, on either wheat straw or seagrass, for 10 days at 30 °C and 110 rpm. For each condition, 18 baffled Erlenmeyers were inoculated with 1 mL of homogenized mycelium from a 5-day-old pre-culture grown on liquid malt medium (malt extract 18 gL^−1^ in water) at 30 °C. Every second day, 3 Erlenmeyers for each condition were sacrificed. All the samples were pooled, and the resulting mycelia were carefully collected with a spatula to separate them from the culture supernatant. The supernatant was pooled for further proteomic analysis. RNA was extracted from the mycelia using the TRIzol reagent (Invitrogen, ThermoFisher Scientific, Madison, WI, USA) according to the manufacturer’s instructions. After purification, total RNA was quantified using a NanoDrop spectrophotometer (ThermoFisher Scientific, Madison, WI, USA) and further purified by LiCl precipitation. RNA samples from all sampling days were pooled to collect the total transcriptome corresponding to the 10-day cultures, and quality was assessed by capillary electrophoresis using a BioAnalyzer (Agilent, Santa Clara, CA, USA).

The pooled RNA sample was sent to Fasteris SA (Geneva, Switzerland) to prepare cDNA libraries and sequence it. Briefly, mRNAs were purified from total RNA using the Dynabeads mRNA purification kit (Invitrogen, ThermoFisher Scientific, Madison, WI, USA), and the DNA library was prepared using the Illumina TruSeq Stranded mRNA Library Prep kit (Illumina, Evry, France). The resulting library was then normalized using the Duplex Specific Nuclease protocol (Evrogen, Euromedex, Souffelweyersheim, France) and sequenced using the Illumina HiSeq-4000 technology (2 × 150 bp) (Illumina, San Diego, CA, USA). Sequencing reads were assembled using the de novo assembler suite of CLCBio Genomic workbench version 11 (https://digitalinsights.qiagen.com), with default parameters for cDNA. Transcripts were converted into open reading frames and proteins by TransDecoder (https://github.com/TransDecoder/TransDecoder). Identical proteins were clustered with a clustering cut-off of 90%.

### 4.5. Preparation of Stemphylium Lucomagnoense Secretomes

For each culture condition (wheat straw or seagrass, with or without sea salt), the supernatants collected for three biological replicates as described in the previous section were centrifuged at 8000 rpm for 10 min at 4 °C and were filtered in three steps on glass microfiber filters through 2.7, 1.6 and 0.7 μm (GF/D, A and F filters, respectively, GE Healthcare Life Sciences, Whatman^TM^, ThermoFisher Scientific, Madison, WI, USA) and then on 0.4 and 0.2 μm PES membranes (Acrodisc^®^, Pall Corporation, Saint-Germain-en-Laye, France). Fifteen milliliters of each culture condition were concentrated three times using 10 kDa cut-off Vivaspin concentrators (Sartorius, Les Ulis, France) and then dialyzed against 50 mM sodium acetate buffer pH 5. The protein concentration in the obtained secretome samples was determined by the Bradford method [63] using bovine serum albumin (BSA) as a standard.

### 4.6. Proteomic Analysis of Secretomes

Short migrations (0.5 cm) of the secretome samples (previous section, 15 μg total protein) were performed by SDS-PAGE on pre-casted polyacrylamide gels (4 to 12% Bis-Tris Mini Gels, Invitrogen, ThermoFisher Scientific, Madison, WI, USA). Gels were stained with Coomassie Brilliant Blue (BioRad, Marnes-la-Coquette, France), and each one-dimensional electrophoresis lane was cut into two (2 mm wide) slices of gel. Protein identification was performed at the PAPPSO platform facility (“Plate-forme d’Analyse Protéomique de Paris Sud-Ouest”, France). In-gel digestion was carried out according to a standard trypsinolysis protocol. Gel pieces were washed twice with 10% acetic acid in 40% ethanol, and then with 50 mM sodium bicarbonate (NH_4_HCO_3_) in 50% acetonitrile (ACN) and incubated in the presence of 10 mM dithiothreitol (DTT) for 30 min at 56 °C. After cooling, the supernatant was removed, and the samples were incubated with 55 mM iodoacetamide at room temperature in the dark for 1 h. Digestion was performed for 8 h at 37 °C with 200 ng of modified trypsin (Promega, Charbonnières-les-Bains, France) dissolved in 50 mM NH_4_CO_3_. Tryptic peptides were first extracted with 50% (*v*/*v*) can, 0.5% (*v*/*v*) trifluoroacetic acid (TFA), and then with pure ACN. Peptide extracts were dried in a vacuum speed concentrator (Thermo Fisher Scientific, Villebon sur Yvette, France) and suspended in 20 μL of 2% (*v*/*v*) ACN and 0.08% (*v*/*v*) TFA. HPLC was performed on an Ultimate 3000 RSLC system (Thermo Fisher Scientific, Madison, WI, USA). Trypsic digestion products were first concentrated and desalted on a pre-column cartridge (PepMap 100 C18, 0.3 × 5 mm, Dionex, Thermo Fisher Scientific, Madison, WI, USA) with 0,08% TFA in 2% ACN at 20 μL/min for 3 min. The pre-column cartridge was connected to the separating column (C18, 0.075 × 500 mm, Thermo Fisher Scientific, Madison, WI, USA), and the peptides were eluted with a linear gradient from 5 to 35% ACN in 0.1% HCOOH for 155 min at 300 nL/min. On-line analysis of peptides was performed with a Tribrid orbitrap fusion lumos mass spectrometer (Thermo Fisher Scientific, Madison, WI, USA), using a nanoelectrospray ion source. Peptide ions were analyzed using Xcalibur 2.1 (Thermo Scientific, Villebon sur Yvette, France) with the following data-dependent acquisition steps: (step 1) full MS scan (mass-to-charge ratio (m/z) 400 to 1400, resolution 70,000) and (step 2) MS/MS (normalized collision energy = 30%, resolution 15,000). The raw mass data were first converted to mzXML format with the MS convert software (ProteoWizard v 3.0.8934) (ProteoWizard, Palo Alto, CA, USA). Protein identification was performed querying MS/MS data against databases, together with an in-house contaminant database, using the X!Tandem pipeline software (version 0.2.34) with the following parameters: One trypsin missed cleavage allowed the alkylation of cysteine and conditional oxidation of methionine, precursor and fragment ion set at 10 ppm and 5 Da, respectively. A refined search was added with similar parameters, except that semi-tryptic peptides, possible N-term acetylation, and histidine mono- and dimethylations were searched. All peptides matched with an E-value lower than 0.05 were parsed with X!Tandem pipeline software. Proteins identified with at least two unique peptides and a log (E-value) lower than −2.6 were validated.

## Figures and Tables

**Table 1:** Summary of assembled contig statistics for de novo transcriptome of marine *Stemphylium lucomagnoense.*

	Assembled Contig Statistics
Total length of contigs	110,739,726 bp
Total number of contigs	350,657
N25 stats	25% of total contig length is contained in the 5614 contigs > = 2983 bp
N50 stats	50% of total contig length is contained in the 26,298 contigs > = 504 bp
N75 stats	75% of total contig length is contained in the 142,056 contigs > = 176 bp
Total GC count	59,818,129 bp
% GC	54.02

**Table 2:** CAZymes identified in *Stemphylium lucomagnoense* secretomes grown on either seagrass or wheat straw, in saline and non-saline conditions.

Accession Number	Number of Total Spectra		Number of Unique Peptides		Predicted Protein Functions	Predicted Family (Subfamily) *	Induction
	W/Ws	S/Ss	W/Ws	S/Ss			
APMZ2_prot13181	222/101	26/13	75/43	16/10	Glucoamylase	GH15-CBM20	S/Ss W/Ws
APMZ2_prot912	110/53	17/6	41/30	14/6	Copper radical oxidase	AA5_1	S/Ss W/Ws
APMZ2_prot15973	92/56	15/10	33/25	9/8	β-1,3-glucanase	GH55	S/Ss W/Ws
APMZ2_prot2472	58/16	10/7	24/13	9/7	α-amylase	GH13_1	S/Ss W/Ws
APMZ2_prot14594	50/0	3/0	31/0	3/0	β-1,4-xylanase	GH10	S W
APMZ2_prot3103	36/8	7/0	15/7	6/0	α-mannosidase	GH47	S W/Ws
APMZ2_prot1893	34/5	2/0	28/5	2/0	α-l-rhamnosidase	GH78	S W/Ws
APMZ2_prot27112	33/16	0/0	23/15	0/0	Glucose/methanol/choline oxidoreductase (GMC)	AA3_2	W/Ws
APMZ2_prot4629	29/18	5/8	20/13	5/6	Pyrroloquinoline quinone-dependent oxidoreductase	AA12	S/Ss W/Ws
APMZ2_prot29015	28/0	0/0	22/0	0/0	α-glucosidase	GH31	W
APMZ2_prot1520	20/0	2/0	18/0	2/0	β-1,4-glucosidase	GH3	S W
APMZ2_prot21697	15/2	0/0	12/2	0/0	β-d-glucosaminidase	GH20	W/Ws
APMZ2_prot29106	15/0	0/0	12/0	0/0	α-glucosidase	GH31	W
APMZ2_prot275	13/0	3/0	10/0	3/0	Chitin deacetylase	CBM18-CE4-CBM18-CBM18	S W
APMZ2_prot24323	12/0	2/0	11/0	2/0	Glucose/methanol/choline oxidoreductase (GMC)	AA3_2	S W
APMZ2_prot2902	12/5	0/0	4/0	0/0	α,α-trehalase	GH37	W/Ws
APMZ2_prot3532	11/0	0/0	9/0	0/0	β-1,4-glucosidase	GH3	W
APMZ2_prot3750	11/0	0/0	9/0	0/0	β-1,4-glucosidase	GH3	W
APMZ2_prot15280	11/0	0/0	10/0	0/0	β-1,4-glucosidase	GH3	W
APMZ2_prot8136	10/3	2/0	8/3	2/0	Rhamnogalacturonyl hydrolase	GH105	S W/Ws
APMZ2_prot26178	9/0	2/0	8/0	2/0	Endo/Exo-β-1,4-glucanase	GH55	W/S
APMZ2_prot15779	9/0	0/0	5/0	0/0	α-mannosidase	GH47	W
APMZ2_prot27204	8/8	0/2	7/7	0/2	Glucoamylase	GH15-CBM20	Ss W/Ws
APMZ2_prot7619	8/0	2/0	8/0	2/0	Feruloyl esterase	CE1	S W
APMZ2_prot18739	8/0	0/0	6/0	0/0	Carbohydrate Binding	CBM50-CBM50-CBM50	W
APMZ2_prot8482	7/4	0/0	7/4	0/0	β-1,3-1,4-glucan endo-1,3- β-glucosidase	GH17	W/Ws
APMZ2_prot17304	7/0	3/0	6/0	3/0	β-1,3-galactanase	GH43_24-CBM35	S W
APMZ2_prot11667	7/4	0/0	7/4	0/0	β-glucuronidase/heparanase	GH79	W/Ws
APMZ2_prot17211	7/0	0/0	7/0	0/0	β-l-arabinofuranosidase	GH142	W
APMZ2_prot21080	6/5	0/0	5/5	0/0	Lytic polysaccharide mono-oxygenase (LPMO)	AA11	W/Ws
APMZ2_prot9402	6/0	0/0	6/0	0/0	α-l-arabinofuranosidase	GH51	W
APMZ2_prot13544	6/0	0/0	3/0	0/0	α-mannosidase	GH47	W
APMZ2_prot21512	5/2	0/0	4/0	0/0	β-d-glucosaminidase	GH20	W
APMZ2_prot23159	5/0	0/0	5/0	0/0	Carbohydrate-binding	CBM50	W
APMZ2_prot27520	5/0	0/0	5/0	0/0	β-1,4-glucosidase	GH1	W
APMZ2_prot7701	4/0	2/2	4/0	2/2	Chitin deacetylase	CE4-CBM18-CBM18	S/Ss W
APMZ2_prot6391	4/0	0/0	4/0	0/0	Lacto-*N*-biosidase	GH136	W
APMZ2_prot20074	4/0	0/0	4/0	0/0	Lacto-*N*-biosidase	GH136	W
APMZ2_prot9251	4/0	0/0	4/0	0/0	Chitinase	GH18	W
APMZ2_prot29026	4/0	0/0	4/0	0/0	Carbohydrate esterase	CE15	W
APMZ2_prot1535	4/0	0/0	3/0	0/0	β-1,3-1,4-glucan endo-1,3-β-glucosidase	GH17	W
APMZ2_prot16320	3/0	3/0	3/0	3/0	α-glucosidase	GH31	S W
APMZ2_prot5721	3/0	0/0	3/0	0/0	α-l-rhamnosidase	GH78	W
APMZ2_prot21603	3/0	0/0	3/0	0/0	α-l-arabinofuranosidase	GH43_26	W
APMZ2_prot411	3/0	0/0	3/0	0/0	β-1,4-xylanase	CBM1-GH10	W
APMZ2_prot2229	3/0	0/0	3/0	0/0	Glucose/methanol/choline oxidoreductase (GMC)	AA3_2	W
APMZ2_prot19857	2/0	0/0	2/0	0/0	Dextranase/isopullulanase	GH49	W
APMZ2_prot23037	2/0	0/0	2/0	0/0	α-l-arabinofuranosidase/β-d-xylosidase/α-l-arabinanase/β-d-galactofuranosidase	GH43_22-GH43_34	W
APMZ2_prot3876	2/0	0/0	2/0	0/0	β-d-glucosaminidase	GH20	W
APMZ2_prot11475	0/6	0/0	0/6	0/0	Glucooligosaccharide oxidase	AA7	Ws
APMZ2_prot21161	0/3	0/3	0/3	0/3	Alginate lyase	PL7_4	Ss Ws

W wheat straw, Ws wheat straw with 3% sea salt, S seagrass, Ss seagrass with 3% sea salt, GH glycoside hydrolase, PL polysaccharide lyase, AA auxiliary activities, CE carbohydrate esterase, CBM carbohydrate-binding module, GMC glucose/methanol/choline oxidoreductase, LPMO lytic polysaccharide mono-oxygenase. * Families were assigned according to the CAZy database.

## Supplementary Materials

The following are available online at https://www.mdpi.com/1660-3397/18/9/461/s1, Table S1: Statistical analysis results.
