# Supplementary material for: Characterization of the CAZy Repertoire from the Marine-Derived Fungus Stemphylium lucomagnoense in Relation to Saline Conditions
Source: Mar Drugs. 2020 Sep 9;18(9):461. doi: 10.3390/md18090461 (PMC7551824; doi:10.3390/md18090461)
Supplement: Supplementary file 1 [file marinedrugs-18-00461-s001.pdf]

SUPPLEMENTARY DATA FOR FIGURE 2

1) ACTIVITY LACCASE

Kruskal-Wallis rank sum test :

Kruskal-Wallis chi-squared = 110.88, df = 19, p-value = 5.481e-15

RESULTS OF THE DUNN PAIRWISE MULTIPLE TEST COMPARISON

Dunn (1964) Kruskal-Wallis multiple comparison p-values adjusted with the Benjamini-Hochberg method

	Comparison	Z	P.unadj	P.adj
1	ASPseagrasslaccasenosalt - ASPseagrasslaccasesalt	0.187679156	8.511282e-01	9.136404e-01
2	ASPseagrasslaccasenosalt - ASPwheatstrawlaccasenosalt	-0.863324118	3.879593e-01	5.191005e-01
3	ASPseagrasslaccasesalt - ASPwheatstrawlaccasenosalt	-1.051003274	2.932571e-01	4.189387e-01
4	ASPseagrasslaccasenosalt - ASPwheatstrawlaccasesalt	0.713180793	4.757339e-01	6.025963e-01
5	ASPseagrasslaccasesalt - ASPwheatstrawlaccasesalt	0.525501637	5.992345e-01	7.028059e-01
6	ASPwheatstrawlaccasenosalt - ASPwheatstrawlaccasesalt	1.576504912	1.149095e-01	2.003009e-01
7	ASPseagrasslaccasenosalt - STYseagrasslaccasenosalt	-1.572334264	1.158731e-01	2.001444e-01
8	ASPseagrasslaccasesalt - STYseagrasslaccasenosalt	-1.760013420	7.840553e-02	1.474956e-01
9	ASPwheatstrawlaccasenosalt - STYseagrasslaccasenosalt	-0.709010145	4.783182e-01	6.018573e-01
10	ASPwheatstrawlaccasesalt - STYseagrasslaccasenosalt	-2.285515057	2.228264e-02	5.962961e-02
11	ASPseagrasslaccasenosalt - STYseagrasslaccasesalt	-2.239637930	2.511444e-02	6.197069e-02
12	ASPseagrasslaccasesalt - STYseagrasslaccasesalt	-2.427317086	1.521095e-02	4.515752e-02
13	ASPwheatstrawlaccasenosalt - STYseagrasslaccasesalt	-1.376313812	1.687245e-01	2.671471e-01
14	ASPwheatstrawlaccasesalt - STYseagrasslaccasesalt	-2.952818723	3.148868e-03	1.272947e-02
15	STYseagrasslaccasenosalt - STYseagrasslaccasesalt	-0.667303666	5.045782e-01	6.106360e-01
16	ASPseagrasslaccasenosalt - STYwheatstrawlaccasenosalt	-2.840211229	4.508367e-03	1.679588e-02
17	ASPseagrasslaccasesalt - STYwheatstrawlaccasenosalt	-3.027890386	2.462674e-03	1.063427e-02
18	ASPwheatstrawlaccasenosalt - STYwheatstrawlaccasenosalt	-1.976887111	4.805439e-02	9.924277e-02
19	ASPwheatstrawlaccasesalt - STYwheatstrawlaccasenosalt	-3.553392023	3.802973e-04	2.189590e-03
20	STYseagrasslaccasenosalt - STYwheatstrawlaccasenosalt	-1.267876966	2.048419e-01	3.113597e-01
21	STYseagrasslaccasesalt - STYwheatstrawlaccasenosalt	-0.600573300	5.481242e-01	6.591367e-01
22	ASPseagrasslaccasenosalt - STYwheatstrawlaccasesalt	-1.313754093	1.889290e-01	2.894879e-01
23	ASPseagrasslaccasesalt - STYwheatstrawlaccasesalt	-1.501433249	1.332435e-01	2.201415e-01
24	ASPwheatstrawlaccasenosalt - STYwheatstrawlaccasesalt	-0.450429975	6.524004e-01	7.467234e-01
25	ASPwheatstrawlaccasesalt - STYwheatstrawlaccasesalt	-2.026934886	4.266907e-02	9.212641e-02
26	STYseagrasslaccasenosalt - STYwheatstrawlaccasesalt	0.258580171	7.959592e-01	8.741748e-01
27	STYseagrasslaccasesalt - STYwheatstrawlaccasesalt	0.925883837	3.545064e-01	4.880885e-01
28	STYwheatstrawlaccasenosalt - STYwheatstrawlaccasesalt	1.526457137	1.268961e-01	2.152701e-01
29	ASPseagrasslaccasenosalt - T1seagrasslaccasenosalt	-2.373098663	1.763955e-02	4.928698e-02
30	ASPseagrasslaccasesalt - T1seagrasslaccasenosalt	-2.560777819	1.044381e-02	3.307207e-02
31	ASPwheatstrawlaccasenosalt - T1seagrasslaccasenosalt	-1.509774545	1.311010e-01	2.185016e-01
32	ASPwheatstrawlaccasesalt - T1seagrasslaccasenosalt	-3.086279456	2.026782e-03	9.168777e-03
33	STYseagrasslaccasenosalt - T1seagrasslaccasenosalt	-0.800764400	4.232681e-01	5.508283e-01
34	STYseagrasslaccasesalt - T1seagrasslaccasenosalt	-0.133460733	8.938290e-01	9.130511e-01
35	STYwheatstrawlaccasenosalt - T1seagrasslaccasenosalt	0.467112566	6.404193e-01	7.419492e-01
36	STYwheatstrawlaccasesalt - T1seagrasslaccasenosalt	-1.059344570	2.894429e-01	4.198027e-01
37	ASPseagrasslaccasenosalt - T1seagrasslaccasesalt	0.008341296	9.933447e-01	9.933447e-01

38	ASPseagrasslaccasesalt - T1seagrasslaccasesalt	-0.179337860	8.576724e-01	9.053209e-01
39	ASPwheatstrawlaccasenosalt - T1seagrasslaccasesalt	0.871665414	3.833909e-01	5.166261e-01
40	ASPwheatstrawlaccasesalt - T1seagrasslaccasesalt	-0.704839497	4.809101e-01	6.011377e-01
41	STYseagrasslaccasenosalt - T1seagrasslaccasesalt	1.580675559	1.139522e-01	2.004715e-01
42	STYseagrasslaccasesalt - T1seagrasslaccasesalt	2.247979226	2.457751e-02	6.144379e-02
43	STYwheatstrawlaccasenosalt - T1seagrasslaccasesalt	2.848552525	4.391860e-03	1.668907e-02
44	STYwheatstrawlaccasesalt - T1seagrasslaccasesalt	1.322095389	1.861364e-01	2.875278e-01
45	T1seagrasslaccasenosalt - T1seagrasslaccasesalt	2.381439959	1.724510e-02	4.890402e-02
46	ASPseagrasslaccasenosalt - T1wheatstrawlaccasenosalt	-4.508470395	6.529668e-06	1.378485e-04
47	ASPseagrasslaccasesalt - T1wheatstrawlaccasenosalt	-4.696149551	2.651115e-06	1.259279e-04
48	ASPwheatstrawlaccasenosalt - T1wheatstrawlaccasenosalt	-3.645146277	2.672398e-04	1.813413e-03
49	ASPwheatstrawlaccasesalt - T1wheatstrawlaccasenosalt	-5.221651188	1.773348e-07	3.369360e-05
50	STYseagrasslaccasenosalt - T1wheatstrawlaccasenosalt	-2.936136132	3.323284e-03	1.315467e-02
51	STYseagrasslaccasesalt - T1wheatstrawlaccasenosalt	-2.268832465	2.327852e-02	5.976916e-02
52	STYwheatstrawlaccasenosalt - T1wheatstrawlaccasenosalt	-1.668259166	9.526429e-02	1.740405e-01
53	STYwheatstrawlaccasesalt - T1wheatstrawlaccasenosalt	-3.194716302	1.399684e-03	6.486338e-03
54	T1seagrasslaccasenosalt - T1wheatstrawlaccasenosalt	-2.135371732	3.273066e-02	7.773531e-02
55	T1seagrasslaccasesalt - T1wheatstrawlaccasenosalt	-4.516811691	6.277769e-06	1.490970e-04
56	ASPseagrasslaccasenosalt - T1wheatstrawlaccasesalt	-3.424101937	6.168348e-04	3.084174e-03
57	ASPseagrasslaccasesalt - T1wheatstrawlaccasesalt	-3.611781094	3.041012e-04	1.863846e-03
58	ASPwheatstrawlaccasenosalt - T1wheatstrawlaccasesalt	-2.560777819	1.044381e-02	3.363262e-02
59	ASPwheatstrawlaccasesalt - T1wheatstrawlaccasesalt	-4.137282731	3.514430e-05	3.338708e-04
60	STYseagrasslaccasenosalt - T1wheatstrawlaccasesalt	-1.851767674	6.405919e-02	1.229419e-01
61	STYseagrasslaccasesalt - T1wheatstrawlaccasesalt	-1.184464008	2.362294e-01	3.534141e-01
62	STYwheatstrawlaccasenosalt - T1wheatstrawlaccasesalt	-0.583890708	5.592938e-01	6.683386e-01
63	STYwheatstrawlaccasesalt - T1wheatstrawlaccasesalt	-2.110347845	3.482841e-02	8.069996e-02
64	T1seagrasslaccasenosalt - T1wheatstrawlaccasesalt	-1.051003274	2.932571e-01	4.221125e-01
65	T1seagrasslaccasesalt - T1wheatstrawlaccasesalt	-3.432443233	5.981691e-04	3.071679e-03
66	T1wheatstrawlaccasenosalt - T1wheatstrawlaccasesalt	1.084368458	2.782015e-01	4.066021e-01
67	ASPseagrasslaccasenosalt - T2seagrasslaccasenosalt	-1.868450266	6.169934e-02	1.208544e-01
68	ASPseagrasslaccasesalt - T2seagrasslaccasenosalt	-2.056129422	3.977004e-02	8.995605e-02
69	ASPwheatstrawlaccasenosalt - T2seagrasslaccasenosalt	-1.005126147	3.148361e-01	4.464094e-01
70	ASPwheatstrawlaccasesalt - T2seagrasslaccasenosalt	-2.581631059	9.833465e-03	3.221307e-02
71	STYseagrasslaccasenosalt - T2seagrasslaccasenosalt	-0.296116002	7.671415e-01	8.474237e-01
72	STYseagrasslaccasesalt - T2seagrasslaccasenosalt	0.371187664	7.104978e-01	7.987845e-01
73	STYwheatstrawlaccasenosalt - T2seagrasslaccasenosalt	0.971760964	3.311695e-01	4.626632e-01
74	STYwheatstrawlaccasesalt - T2seagrasslaccasenosalt	-0.554696173	5.791025e-01	6.876842e-01
75	T1seagrasslaccasenosalt - T2seagrasslaccasenosalt	0.504648398	6.138058e-01	7.154792e-01
76	T1seagrasslaccasesalt - T2seagrasslaccasenosalt	-1.876791561	6.054667e-02	1.198319e-01
77	T1wheatstrawlaccasenosalt - T2seagrasslaccasenosalt	2.640020130	8.290110e-03	2.763370e-02
78	T1wheatstrawlaccasesalt - T2seagrasslaccasenosalt	1.555651672	1.197909e-01	2.050476e-01
79	ASPseagrasslaccasenosalt - T2seagrasslaccasesalt	-0.145972677	8.839430e-01	9.127672e-01
80	ASPseagrasslaccasesalt - T2seagrasslaccasesalt	-0.333651833	7.386423e-01	8.207137e-01
81	ASPwheatstrawlaccasenosalt - T2seagrasslaccasesalt	0.717351441	4.731573e-01	6.033549e-01
82	ASPwheatstrawlaccasesalt - T2seagrasslaccasesalt	-0.859153470	3.902559e-01	5.185218e-01
83	STYseagrasslaccasenosalt - T2seagrasslaccasesalt	1.426361587	1.537640e-01	2.475861e-01
84	STYseagrasslaccasesalt - T2seagrasslaccasesalt	2.093665253	3.628981e-02	8.307306e-02
85	STYwheatstrawlaccasenosalt - T2seagrasslaccasesalt	2.694238552	7.054965e-03	2.393649e-02
86	STYwheatstrawlaccasesalt - T2seagrasslaccasesalt	1.167781416	2.428949e-01	3.605472e-01

87	T1seagrasslaccasenosalt - T2seagrasslaccasesalt	2.227125986	2.593885e-02	6.318439e-02
88	T1seagrasslaccasesalt - T2seagrasslaccasesalt	-0.154313973	8.773622e-01	9.159276e-01
89	T1wheatstrawlaccasenosalt - T2seagrasslaccasesalt	4.362497718	1.285859e-05	2.035944e-04
90	T1wheatstrawlaccasesalt - T2seagrasslaccasesalt	3.278129260	1.044975e-03	4.963633e-03
91	T2seagrasslaccasenosalt - T2seagrasslaccasesalt	1.722477589	8.498304e-02	1.567648e-01
92	ASPseagrasslaccasenosalt - T2wheatstrawlaccasenosalt	-4.283255408	1.841785e-05	1.944107e-04
93	ASPseagrasslaccasesalt - T2wheatstrawlaccasenosalt	-4.470934564	7.787853e-06	1.479692e-04
94	ASPwheatstrawlaccasenosalt - T2wheatstrawlaccasenosalt	-3.419931290	6.263695e-04	3.051544e-03
95	ASPwheatstrawlaccasesalt - T2wheatstrawlaccasenosalt	-4.996436201	5.839948e-07	3.698634e-05
96	STYseagrasslaccasenosalt - T2wheatstrawlaccasenosalt	-2.710921144	6.709658e-03	2.317882e-02
97	STYseagrasslaccasesalt - T2wheatstrawlaccasenosalt	-2.043617478	4.099135e-02	9.056229e-02
98	STYwheatstrawlaccasenosalt - T2wheatstrawlaccasenosalt	-1.443044178	1.490080e-01	2.419788e-01
99	STYwheatstrawlaccasesalt - T2wheatstrawlaccasenosalt	-2.969501315	2.982835e-03	1.259419e-02
100	T1seagrasslaccasenosalt - T2wheatstrawlaccasenosalt	-1.910156745	5.611303e-02	1.134200e-01
101	T1seagrasslaccasesalt - T2wheatstrawlaccasenosalt	-4.291596704	1.773929e-05	1.982627e-04
102	T1wheatstrawlaccasenosalt - T2wheatstrawlaccasenosalt	0.225214987	8.218120e-01	8.922531e-01
103	T1wheatstrawlaccasesalt - T2wheatstrawlaccasenosalt	-0.859153470	3.902559e-01	5.149209e-01
104	T2seagrasslaccasenosalt - T2wheatstrawlaccasenosalt	-2.414805142	1.574363e-02	4.601984e-02
105	T2seagrasslaccasesalt - T2wheatstrawlaccasenosalt	-4.137282731	3.514430e-05	3.179722e-04
106	ASPseagrasslaccasenosalt - T2wheatstrawlaccasesalt	-3.824484137	1.310462e-04	9.959510e-04
107	ASPseagrasslaccasesalt - T2wheatstrawlaccasesalt	-4.012163293	6.016486e-05	5.196056e-04
108	ASPwheatstrawlaccasenosalt - T2wheatstrawlaccasesalt	-2.961160019	3.064827e-03	1.265907e-02
109	ASPwheatstrawlaccasesalt - T2wheatstrawlaccasesalt	-4.537664931	5.688054e-06	1.543900e-04
110	STYseagrasslaccasenosalt - T2wheatstrawlaccasesalt	-2.252149874	2.431280e-02	6.159243e-02
111	STYseagrasslaccasesalt - T2wheatstrawlaccasesalt	-1.584846207	1.130013e-01	2.006565e-01
112	STYwheatstrawlaccasenosalt - T2wheatstrawlaccasesalt	-0.984272908	3.249813e-01	4.573812e-01
113	STYwheatstrawlaccasesalt - T2wheatstrawlaccasesalt	-2.510730044	1.204818e-02	3.752712e-02
114	T1seagrasslaccasenosalt - T2wheatstrawlaccasesalt	-1.451385474	1.466726e-01	2.402395e-01
115	T1seagrasslaccasesalt - T2wheatstrawlaccasesalt	-3.832825433	1.266799e-04	1.002882e-03
116	T1wheatstrawlaccasenosalt - T2wheatstrawlaccasesalt	0.683986258	4.939838e-01	6.094606e-01
117	T1wheatstrawlaccasesalt - T2wheatstrawlaccasesalt	-0.400382200	6.888750e-01	7.790849e-01
118	T2seagrasslaccasenosalt - T2wheatstrawlaccasesalt	-1.956033872	5.046116e-02	1.030927e-01
119	T2seagrasslaccasesalt - T2wheatstrawlaccasesalt	-3.678511460	2.345992e-04	1.650883e-03
120	T2wheatstrawlaccasenosalt - T2wheatstrawlaccasesalt	0.458771271	6.463984e-01	7.443376e-01
121	ASPseagrasslaccasenosalt - T3seagrasslaccasenosalt	-2.014422942	4.396515e-02	9.281531e-02
122	ASPseagrasslaccasesalt - T3seagrasslaccasenosalt	-2.202102099	2.765810e-02	6.651947e-02
123	ASPwheatstrawlaccasenosalt - T3seagrasslaccasenosalt	-1.151098824	2.496916e-01	3.677628e-01
124	ASPwheatstrawlaccasesalt - T3seagrasslaccasenosalt	-2.727603736	6.379619e-03	2.244681e-02
125	STYseagrasslaccasenosalt - T3seagrasslaccasenosalt	-0.442088679	6.584250e-01	7.491063e-01
126	STYseagrasslaccasesalt - T3seagrasslaccasenosalt	0.225214987	8.218120e-01	8.973810e-01
127	STYwheatstrawlaccasenosalt - T3seagrasslaccasenosalt	0.825788287	4.089242e-01	5.358317e-01
128	STYwheatstrawlaccasesalt - T3seagrasslaccasenosalt	-0.700668850	4.835097e-01	6.004369e-01
129	T1seagrasslaccasenosalt - T3seagrasslaccasenosalt	0.358675721	7.198377e-01	8.045245e-01
130	T1seagrasslaccasesalt - T3seagrasslaccasenosalt	-2.022764238	4.309746e-02	9.200581e-02
131	T1wheatstrawlaccasenosalt - T3seagrasslaccasenosalt	2.494047453	1.262957e-02	3.870351e-02
132	T1wheatstrawlaccasesalt - T3seagrasslaccasenosalt	1.409678995	1.586345e-01	2.532820e-01
133	T2seagrasslaccasenosalt - T3seagrasslaccasenosalt	-0.145972677	8.839430e-01	9.078333e-01
134	T2seagrasslaccasesalt - T3seagrasslaccasenosalt	-1.868450266	6.169934e-02	1.196212e-01
135	T2wheatstrawlaccasenosalt - T3seagrasslaccasenosalt	2.268832465	2.327852e-02	6.058792e-02

136 T2wheatstrawlaccasesalt - T3seagrasslaccasenosalt 1.810061195 7.028630e-02 1.335440e-01
137 ASPseagrasslaccasenosalt - T3seagrasslaccasesalt 0.033365183 9.733834e-01 9.837385e-01
138 ASPseagrasslaccasesalt - T3seagrasslaccasesalt -0.154313973 8.773622e-01 9.109225e-01
139 ASPwheatstrawlaccasenosalt - T3seagrasslaccasesalt 0.896689302 3.698847e-01 5.019864e-01
140 ASPwheatstrawlaccasesalt - T3seagrasslaccasesalt -0.679815610 4.966212e-01 6.087615e-01
141 STYseagrasslaccasenosalt - T3seagrasslaccasesalt 1.605699447 1.083400e-01 1.960437e-01
142 STYseagrasslaccasesalt - T3seagrasslaccasesalt 2.273003113 2.302599e-02 6.076304e-02
143 STYwheatstrawlaccasenosalt - T3seagrasslaccasesalt 2.873576413 4.058529e-03 1.573715e-02
144 STYwheatstrawlaccasesalt - T3seagrasslaccasesalt 1.347119276 1.779418e-01 2.771225e-01
145 T1seagrasslaccasenosalt - T3seagrasslaccasesalt 2.406463846 1.610780e-02 4.637093e-02
146 T1seagrasslaccasesalt - T3seagrasslaccasesalt 0.025023887 9.800359e-01 9.852213e-01
147 T1wheatstrawlaccasenosalt - T3seagrasslaccasesalt 4.541835578 5.576653e-06 2.119128e-04
148 T1wheatstrawlaccasesalt - T3seagrasslaccasesalt 3.457467121 5.452788e-04 2.877860e-03
149 T2seagrasslaccasenosalt - T3seagrasslaccasesalt 1.901815449 5.719529e-02 1.143906e-01
150 T2seagrasslaccasesalt - T3seagrasslaccasesalt 0.179337860 8.576724e-01 9.103785e-01
151 T2wheatstrawlaccasenosalt - T3seagrasslaccasesalt 4.316620591 1.584361e-05 1.881429e-04
152 T2wheatstrawlaccasesalt - T3seagrasslaccasesalt 3.857849321 1.143891e-04 9.449537e-04
153 T3seagrasslaccasenosalt - T3seagrasslaccasesalt 2.047788126 4.058076e-02 9.070994e-02
154 ASPseagrasslaccasenosalt - T3wheatstrawlaccasenosalt -4.349985774 1.361464e-05 1.847701e-04
155 ASPseagrasslaccasesalt - T3wheatstrawlaccasenosalt -4.537664931 5.688054e-06 1.801217e-04
156 ASPwheatstrawlaccasenosalt - T3wheatstrawlaccasenosalt -3.486661656 4.890897e-04 2.733148e-03
157 ASPwheatstrawlaccasesalt - T3wheatstrawlaccasenosalt -5.063166568 4.123493e-07 3.917318e-05
158 STYseagrasslaccasenosalt - T3wheatstrawlaccasenosalt -2.777651511 5.475331e-03 2.000602e-02
159 STYseagrasslaccasesalt - T3wheatstrawlaccasenosalt -2.110347845 3.482841e-02 8.169626e-02
160 STYwheatstrawlaccasenosalt - T3wheatstrawlaccasenosalt -1.509774545 1.311010e-01 2.204352e-01
161 STYwheatstrawlaccasesalt - T3wheatstrawlaccasenosalt -3.036231681 2.395552e-03 1.058500e-02
162 T1seagrasslaccasenosalt - T3wheatstrawlaccasenosalt -1.976887111 4.805439e-02 1.003333e-01
163 T1seagrasslaccasesalt - T3wheatstrawlaccasenosalt -4.358327070 1.310604e-05 1.915498e-04
164 T1wheatstrawlaccasenosalt - T3wheatstrawlaccasenosalt 0.158484621 8.740749e-01 9.175372e-01
165 T1wheatstrawlaccasesalt - T3wheatstrawlaccasenosalt -0.925883837 3.545064e-01 4.916511e-01
166 T2seagrasslaccasenosalt - T3wheatstrawlaccasenosalt -2.481535509 1.308177e-02 3.945295e-02
167 T2seagrasslaccasesalt - T3wheatstrawlaccasenosalt -4.204013097 2.622238e-05 2.622238e-04
168 T2wheatstrawlaccasenosalt - T3wheatstrawlaccasenosalt -0.066730367 9.467964e-01 9.619856e-01
169 T2wheatstrawlaccasesalt - T3wheatstrawlaccasenosalt -0.525501637 5.992345e-01 7.071712e-01
170 T3seagrasslaccasenosalt - T3wheatstrawlaccasenosalt -2.335562832 1.951404e-02 5.373431e-02
171 T3seagrasslaccasesalt - T3wheatstrawlaccasenosalt -4.383350958 1.168676e-05 2.018622e-04
172 ASPseagrasslaccasenosalt - T3wheatstrawlaccasesalt -3.607610446 3.090300e-04 1.834866e-03
173 ASPseagrasslaccasesalt - T3wheatstrawlaccasesalt -3.795289602 1.474712e-04 1.077674e-03
174 ASPwheatstrawlaccasenosalt - T3wheatstrawlaccasesalt -2.744286327 6.064262e-03 2.173981e-02
175 ASPwheatstrawlaccasesalt - T3wheatstrawlaccasesalt -4.320791239 1.554707e-05 1.969296e-04
176 STYseagrasslaccasenosalt - T3wheatstrawlaccasesalt -2.035276182 4.182309e-02 9.133779e-02
177 STYseagrasslaccasesalt - T3wheatstrawlaccasesalt -1.367972516 1.713207e-01 2.690159e-01
178 STYwheatstrawlaccasenosalt - T3wheatstrawlaccasesalt -0.767399216 4.428442e-01 5.723837e-01
179 STYwheatstrawlaccasesalt - T3wheatstrawlaccasesalt -2.293856353 2.179875e-02 5.916804e-02
180 T1seagrasslaccasenosalt - T3wheatstrawlaccasesalt -1.234511783 2.170123e-01 3.272407e-01
181 T1seagrasslaccasesalt - T3wheatstrawlaccasesalt -3.615951742 2.992461e-04 1.895225e-03
182 T1wheatstrawlaccasenosalt - T3wheatstrawlaccasesalt 0.900859949 3.676628e-01 5.025606e-01
183 T1wheatstrawlaccasesalt - T3wheatstrawlaccasesalt -0.183508508 8.543990e-01 9.119990e-01
184 T2seagrasslaccasenosalt - T3wheatstrawlaccasesalt -1.739160180 8.200659e-02 1.527574e-01

```
185        T2seagrasslaccasesalt - T3wheatstrawlaccasesalt -3.461637769 5.368992e-04 2.914596e-03
186    T2wheatstrawlaccasenosalt - T3wheatstrawlaccasesalt  0.675644962 4.992661e-01 6.080805e-01
187      T2wheatstrawlaccasesalt - T3wheatstrawlaccasesalt  0.216873692 8.283068e-01 8.941948e-01
188      T3seagrasslaccasenosalt - T3wheatstrawlaccasesalt -1.593187503 1.111181e-01 1.991740e-01
189        T3seagrasslaccasesalt - T3wheatstrawlaccasesalt -3.640975629 2.716068e-04 1.779493e-03
190    T3wheatstrawlaccasenosalt - T3wheatstrawlaccasesalt  0.742375329 4.578600e-01 5.877932e-01
> library(rcompanion)
> cldList(P.adj ~ Comparison,data=PT, threshold+0,05)
                         Group  Letter MonoLetter
1    ASPseagrasslaccasenosalt     abc  abc
2      ASPseagrasslaccasesalt      ab  ab
3  ASPwheatstrawlaccasenosalt   abcde  abcde
4    ASPwheatstrawlaccasesalt       a  a
5    STYseagrasslaccasenosalt abcdefg  abcdefg
6      STYseagrasslaccasesalt cdefghi    cdefghi
7  STYwheatstrawlaccasenosalt   dfghi     d fghi
8    STYwheatstrawlaccasesalt  abcdef  abcdef
9     T1seagrasslaccasenosalt  defghi     defghi
10      T1seagrasslaccasesalt     abc  abc
11  T1wheatstrawlaccasenosalt       h        h
12    T1wheatstrawlaccasesalt    fghi      fghi
13    T2seagrasslaccasenosalt  bcdefg   bcdefg
14      T2seagrasslaccasesalt    abce  abc e
15  T2wheatstrawlaccasenosalt      hi        hi
16    T2wheatstrawlaccasesalt     ghi       ghi
17    T3seagrasslaccasenosalt bcdefgi   bcdefg i
18      T3seagrasslaccasesalt     abc  abc
19  T3wheatstrawlaccasenosalt      hi        hi
20    T3wheatstrawlaccasesalt    fghi      fghi
> R<-
dunn.test(activite_laccase$value,activite_laccase$groupe,method="bonferroni",kw=TRUE,label=TRUE,wrap=TRU
E,table=FALSE,list=TRUE)
  Kruskal-Wallis rank sum test

data: x and group
Kruskal-Wallis chi-squared = 110.8824, df = 19, p-value = 0


                           Comparison of x by group
                                 (Bonferroni)

List of pairwise comparisons: Z statistic (adjusted p-value)
-----------------------------------------------------------------------------
ASPseagrasslaccasenosalt - ASPseagrasslaccasesalt       :  0.187679 (1.0000)
ASPseagrasslaccasenosalt - ASPwheatstrawlaccasenosalt   : -0.863324 (1.0000)
ASPseagrasslaccasesalt - ASPwheatstrawlaccasenosalt     : -1.051003 (1.0000)
ASPseagrasslaccasenosalt - ASPwheatstrawlaccasesalt     :  0.713180 (1.0000)
ASPseagrasslaccasesalt - ASPwheatstrawlaccasesalt       :  0.525501 (1.0000)
ASPwheatstrawlaccasenosalt - ASPwheatstrawlaccasesalt   :  1.576504 (1.0000)
ASPseagrasslaccasenosalt - STYseagrasslaccasenosalt     : -1.572334 (1.0000)
```

ASPseagrasslaccasesalt - STYseagrasslaccasenosalt : -1.760013 (1.0000)
ASPwheatstrawlaccasenosalt - STYseagrasslaccasenosalt : -0.709010 (1.0000)
ASPwheatstrawlaccasesalt - STYseagrasslaccasenosalt : -2.285515 (1.0000)
ASPseagrasslaccasenosalt - STYseagrasslaccasesalt : -2.239637 (1.0000)
ASPseagrasslaccasesalt - STYseagrasslaccasesalt : -2.427317 (1.0000)
ASPwheatstrawlaccasenosalt - STYseagrasslaccasesalt : -1.376313 (1.0000)
ASPwheatstrawlaccasesalt - STYseagrasslaccasesalt : -2.952818 (0.2991)
STYseagrasslaccasenosalt - STYseagrasslaccasesalt : -0.667303 (1.0000)
ASPseagrasslaccasenosalt - STYwheatstrawlaccasenosalt : -2.840211 (0.4283)
ASPseagrasslaccasesalt - STYwheatstrawlaccasenosalt : -3.027890 (0.2340)
ASPwheatstrawlaccasenosalt - STYwheatstrawlaccasenosalt : -1.976887 (1.0000)
ASPwheatstrawlaccasesalt - STYwheatstrawlaccasenosalt : -3.553392 (0.0361)
STYseagrasslaccasenosalt - STYwheatstrawlaccasenosalt : -1.267876 (1.0000)
STYseagrasslaccasesalt - STYwheatstrawlaccasenosalt : -0.600573 (1.0000)
ASPseagrasslaccasenosalt - STYwheatstrawlaccasesalt : -1.313754 (1.0000)
ASPseagrasslaccasesalt - STYwheatstrawlaccasesalt : -1.501433 (1.0000)
ASPwheatstrawlaccasenosalt - STYwheatstrawlaccasesalt : -0.450429 (1.0000)
ASPwheatstrawlaccasesalt - STYwheatstrawlaccasesalt : -2.026934 (1.0000)
STYseagrasslaccasenosalt - STYwheatstrawlaccasesalt : 0.258580 (1.0000)
STYseagrasslaccasesalt - STYwheatstrawlaccasesalt : 0.925883 (1.0000)
STYwheatstrawlaccasenosalt - STYwheatstrawlaccasesalt : 1.526457 (1.0000)
ASPseagrasslaccasenosalt - T1seagrasslaccasenosalt : -2.373098 (1.0000)
ASPseagrasslaccasesalt - T1seagrasslaccasenosalt : -2.560777 (0.9922)
ASPwheatstrawlaccasenosalt - T1seagrasslaccasenosalt : -1.509774 (1.0000)
ASPwheatstrawlaccasesalt - T1seagrasslaccasenosalt : -3.086279 (0.1925)
STYseagrasslaccasenosalt - T1seagrasslaccasenosalt : -0.800764 (1.0000)
STYseagrasslaccasesalt - T1seagrasslaccasenosalt : -0.133460 (1.0000)
STYwheatstrawlaccasenosalt - T1seagrasslaccasenosalt : 0.467112 (1.0000)
STYwheatstrawlaccasesalt - T1seagrasslaccasenosalt : -1.059344 (1.0000)
ASPseagrasslaccasenosalt - T1seagrasslaccasesalt : 0.008341 (1.0000)
ASPseagrasslaccasesalt - T1seagrasslaccasesalt : -0.179337 (1.0000)
ASPwheatstrawlaccasenosalt - T1seagrasslaccasesalt : 0.871665 (1.0000)
ASPwheatstrawlaccasesalt - T1seagrasslaccasesalt : -0.704839 (1.0000)
STYseagrasslaccasenosalt - T1seagrasslaccasesalt : 1.580675 (1.0000)
STYseagrasslaccasesalt - T1seagrasslaccasesalt : 2.247979 (1.0000)
STYwheatstrawlaccasenosalt - T1seagrasslaccasesalt : 2.848552 (0.4172)
STYwheatstrawlaccasesalt - T1seagrasslaccasesalt : 1.322095 (1.0000)
T1seagrasslaccasenosalt - T1seagrasslaccasesalt : 2.381439 (1.0000)
ASPseagrasslaccasenosalt - T1wheatstrawlaccasenosalt : -4.508470 (0.0006)*
ASPseagrasslaccasesalt - T1wheatstrawlaccasenosalt : -4.696149 (0.0003)*
ASPwheatstrawlaccasenosalt - T1wheatstrawlaccasenosalt : -3.645146 (0.0254)
ASPwheatstrawlaccasesalt - T1wheatstrawlaccasenosalt : -5.221651 (0.0000)*
STYseagrasslaccasenosalt - T1wheatstrawlaccasenosalt : -2.936136 (0.3157)
STYseagrasslaccasesalt - T1wheatstrawlaccasenosalt : -2.268832 (1.0000)
STYwheatstrawlaccasenosalt - T1wheatstrawlaccasenosalt : -1.668259 (1.0000)
STYwheatstrawlaccasesalt - T1wheatstrawlaccasenosalt : -3.194716 (0.1330)
T1seagrasslaccasenosalt - T1wheatstrawlaccasenosalt : -2.135371 (1.0000)
T1seagrasslaccasesalt - T1wheatstrawlaccasenosalt : -4.516811 (0.0006)*
ASPseagrasslaccasenosalt - T1wheatstrawlaccasesalt : -3.424101 (0.0586)

ASPseagrasslaccasesalt - T1wheatstrawlaccasesalt : -3.611781 (0.0289)
ASPwheatstrawlaccasenosalt - T1wheatstrawlaccasesalt : -2.560777 (0.9922)
ASPwheatstrawlaccasesalt - T1wheatstrawlaccasesalt : -4.137282 (0.0033)*
STYseagrasslaccasenosalt - T1wheatstrawlaccasesalt : -1.851767 (1.0000)
STYseagrasslaccasesalt - T1wheatstrawlaccasesalt : -1.184464 (1.0000)
STYwheatstrawlaccasenosalt - T1wheatstrawlaccasesalt : -0.583890 (1.0000)
STYwheatstrawlaccasesalt - T1wheatstrawlaccasesalt : -2.110347 (1.0000)
T1seagrasslaccasenosalt - T1wheatstrawlaccasesalt : -1.051003 (1.0000)
T1seagrasslaccasesalt - T1wheatstrawlaccasesalt : -3.432443 (0.0568)
T1wheatstrawlaccasenosalt - T1wheatstrawlaccasesalt : 1.084368 (1.0000)
ASPseagrasslaccasenosalt - T2seagrasslaccasenosalt : -1.868450 (1.0000)
ASPseagrasslaccasesalt - T2seagrasslaccasenosalt : -2.056129 (1.0000)
ASPwheatstrawlaccasenosalt - T2seagrasslaccasenosalt : -1.005126 (1.0000)
ASPwheatstrawlaccasesalt - T2seagrasslaccasenosalt : -2.581631 (0.9342)
STYseagrasslaccasenosalt - T2seagrasslaccasenosalt : -0.296116 (1.0000)
STYseagrasslaccasesalt - T2seagrasslaccasenosalt : 0.371187 (1.0000)
STYwheatstrawlaccasenosalt - T2seagrasslaccasenosalt : 0.971760 (1.0000)
STYwheatstrawlaccasesalt - T2seagrasslaccasenosalt : -0.554696 (1.0000)
T1seagrasslaccasenosalt - T2seagrasslaccasenosalt : 0.504648 (1.0000)
T1seagrasslaccasesalt - T2seagrasslaccasenosalt : -1.876791 (1.0000)
T1wheatstrawlaccasenosalt - T2seagrasslaccasenosalt : 2.640020 (0.7876)
T1wheatstrawlaccasesalt - T2seagrasslaccasenosalt : 1.555651 (1.0000)
ASPseagrasslaccasenosalt - T2seagrasslaccasesalt : -0.145972 (1.0000)
ASPseagrasslaccasesalt - T2seagrasslaccasesalt : -0.333651 (1.0000)
ASPwheatstrawlaccasenosalt - T2seagrasslaccasesalt : 0.717351 (1.0000)
ASPwheatstrawlaccasesalt - T2seagrasslaccasesalt : -0.859153 (1.0000)
STYseagrasslaccasenosalt - T2seagrasslaccasesalt : 1.426361 (1.0000)
STYseagrasslaccasesalt - T2seagrasslaccasesalt : 2.093665 (1.0000)
STYwheatstrawlaccasenosalt - T2seagrasslaccasesalt : 2.694238 (0.6702)
STYwheatstrawlaccasesalt - T2seagrasslaccasesalt : 1.167781 (1.0000)
T1seagrasslaccasenosalt - T2seagrasslaccasesalt : 2.227125 (1.0000)
T1seagrasslaccasesalt - T2seagrasslaccasesalt : -0.154313 (1.0000)
T1wheatstrawlaccasenosalt - T2seagrasslaccasesalt : 4.362497 (0.0012)*
T1wheatstrawlaccasesalt - T2seagrasslaccasesalt : 3.278129 (0.0993)
T2seagrasslaccasenosalt - T2seagrasslaccasesalt : 1.722477 (1.0000)
ASPseagrasslaccasenosalt - T2wheatstrawlaccasenosalt : -4.283255 (0.0017)*
ASPseagrasslaccasesalt - T2wheatstrawlaccasenosalt : -4.470934 (0.0007)*
ASPwheatstrawlaccasenosalt - T2wheatstrawlaccasenosalt : -3.419931 (0.0595)
ASPwheatstrawlaccasesalt - T2wheatstrawlaccasenosalt : -4.996436 (0.0001)*
STYseagrasslaccasenosalt - T2wheatstrawlaccasenosalt : -2.710921 (0.6374)
STYseagrasslaccasesalt - T2wheatstrawlaccasenosalt : -2.043617 (1.0000)
STYwheatstrawlaccasenosalt - T2wheatstrawlaccasenosalt : -1.443044 (1.0000)
STYwheatstrawlaccasesalt - T2wheatstrawlaccasenosalt : -2.969501 (0.2834)
T1seagrasslaccasenosalt - T2wheatstrawlaccasenosalt : -1.910156 (1.0000)
T1seagrasslaccasesalt - T2wheatstrawlaccasenosalt : -4.291596 (0.0017)*
T1wheatstrawlaccasenosalt - T2wheatstrawlaccasenosalt : 0.225214 (1.0000)
T1wheatstrawlaccasesalt - T2wheatstrawlaccasenosalt : -0.859153 (1.0000)
T2seagrasslaccasenosalt - T2wheatstrawlaccasenosalt : -2.414805 (1.0000)
T2seagrasslaccasesalt - T2wheatstrawlaccasenosalt : -4.137282 (0.0033)*

ASPseagrasslaccasenosalt - T2wheatstrawlaccasesalt : -3.824484 (0.0124)*
ASPseagrasslaccasesalt - T2wheatstrawlaccasesalt : -4.012163 (0.0057)*
ASPwheatstrawlaccasenosalt - T2wheatstrawlaccasesalt : -2.961160 (0.2912)
ASPwheatstrawlaccasesalt - T2wheatstrawlaccasesalt : -4.537664 (0.0005)*
STYseagrasslaccasenosalt - T2wheatstrawlaccasesalt : -2.252149 (1.0000)
STYseagrasslaccasesalt - T2wheatstrawlaccasesalt : -1.584846 (1.0000)
STYwheatstrawlaccasenosalt - T2wheatstrawlaccasesalt : -0.984272 (1.0000)
STYwheatstrawlaccasesalt - T2wheatstrawlaccasesalt : -2.510730 (1.0000)
T1seagrasslaccasenosalt - T2wheatstrawlaccasesalt : -1.451385 (1.0000)
T1seagrasslaccasesalt - T2wheatstrawlaccasesalt : -3.832825 (0.0120)*
T1wheatstrawlaccasenosalt - T2wheatstrawlaccasesalt : 0.683986 (1.0000)
T1wheatstrawlaccasesalt - T2wheatstrawlaccasesalt : -0.400382 (1.0000)
T2seagrasslaccasenosalt - T2wheatstrawlaccasesalt : -1.956033 (1.0000)
T2seagrasslaccasesalt - T2wheatstrawlaccasesalt : -3.678511 (0.0223)*
T2wheatstrawlaccasenosalt - T2wheatstrawlaccasesalt : 0.458771 (1.0000)
ASPseagrasslaccasenosalt - T3seagrasslaccasenosalt : -2.014422 (1.0000)
ASPseagrasslaccasesalt - T3seagrasslaccasenosalt : -2.202102 (1.0000)
ASPwheatstrawlaccasenosalt - T3seagrasslaccasenosalt : -1.151098 (1.0000)
ASPwheatstrawlaccasesalt - T3seagrasslaccasenosalt : -2.727603 (0.6061)
STYseagrasslaccasenosalt - T3seagrasslaccasenosalt : -0.442088 (1.0000)
STYseagrasslaccasesalt - T3seagrasslaccasenosalt : 0.225214 (1.0000)
STYwheatstrawlaccasenosalt - T3seagrasslaccasenosalt : 0.825788 (1.0000)
STYwheatstrawlaccasesalt - T3seagrasslaccasenosalt : -0.700668 (1.0000)
T1seagrasslaccasenosalt - T3seagrasslaccasenosalt : 0.358675 (1.0000)
T1seagrasslaccasesalt - T3seagrasslaccasenosalt : -2.022764 (1.0000)
T1wheatstrawlaccasenosalt - T3seagrasslaccasenosalt : 2.494047 (1.0000)
T1wheatstrawlaccasesalt - T3seagrasslaccasenosalt : 1.409678 (1.0000)
T2seagrasslaccasenosalt - T3seagrasslaccasenosalt : -0.145972 (1.0000)
T2seagrasslaccasesalt - T3seagrasslaccasenosalt : -1.868450 (1.0000)
T2wheatstrawlaccasenosalt - T3seagrasslaccasenosalt : 2.268832 (1.0000)
T2wheatstrawlaccasesalt - T3seagrasslaccasenosalt : 1.810061 (1.0000)
ASPseagrasslaccasenosalt - T3seagrasslaccasesalt : 0.033365 (1.0000)
ASPseagrasslaccasesalt - T3seagrasslaccasesalt : -0.154313 (1.0000)
ASPwheatstrawlaccasenosalt - T3seagrasslaccasesalt : 0.896689 (1.0000)
ASPwheatstrawlaccasesalt - T3seagrasslaccasesalt : -0.679815 (1.0000)
STYseagrasslaccasenosalt - T3seagrasslaccasesalt : 1.605699 (1.0000)
STYseagrasslaccasesalt - T3seagrasslaccasesalt : 2.273003 (1.0000)
STYwheatstrawlaccasenosalt - T3seagrasslaccasesalt : 2.873576 (0.3856)
STYwheatstrawlaccasesalt - T3seagrasslaccasesalt : 1.347119 (1.0000)
T1seagrasslaccasenosalt - T3seagrasslaccasesalt : 2.406463 (1.0000)
T1seagrasslaccasesalt - T3seagrasslaccasesalt : 0.025023 (1.0000)
T1wheatstrawlaccasenosalt - T3seagrasslaccasesalt : 4.541835 (0.0005)*
T1wheatstrawlaccasesalt - T3seagrasslaccasesalt : 3.457467 (0.0518)
T2seagrasslaccasenosalt - T3seagrasslaccasesalt : 1.901815 (1.0000)
T2seagrasslaccasesalt - T3seagrasslaccasesalt : 0.179337 (1.0000)
T2wheatstrawlaccasenosalt - T3seagrasslaccasesalt : 4.316620 (0.0015)*
T2wheatstrawlaccasesalt - T3seagrasslaccasesalt : 3.857849 (0.0109)*
T3seagrasslaccasenosalt - T3seagrasslaccasesalt : 2.047788 (1.0000)
ASPseagrasslaccasenosalt - T3wheatstrawlaccasenosalt : -4.349985 (0.0013)*

ASPseagrasslaccasesalt - T3wheatstrawlaccasenosalt : -4.537664 (0.0005)*
ASPwheatstrawlaccasenosalt - T3wheatstrawlaccasenosalt : -3.486661 (0.0465)
ASPwheatstrawlaccasesalt - T3wheatstrawlaccasenosalt : -5.063166 (0.0000)*
STYseagrasslaccasenosalt - T3wheatstrawlaccasenosalt : -2.777651 (0.5202)
STYseagrasslaccasesalt - T3wheatstrawlaccasenosalt : -2.110347 (1.0000)
STYwheatstrawlaccasenosalt - T3wheatstrawlaccasenosalt : -1.509774 (1.0000)
STYwheatstrawlaccasesalt - T3wheatstrawlaccasenosalt : -3.036231 (0.2276)
T1seagrasslaccasenosalt - T3wheatstrawlaccasenosalt : -1.976887 (1.0000)
T1seagrasslaccasesalt - T3wheatstrawlaccasenosalt : -4.358327 (0.0012)*
T1wheatstrawlaccasenosalt - T3wheatstrawlaccasenosalt : 0.158484 (1.0000)
T1wheatstrawlaccasesalt - T3wheatstrawlaccasenosalt : -0.925883 (1.0000)
T2seagrasslaccasenosalt - T3wheatstrawlaccasenosalt : -2.481535 (1.0000)
T2seagrasslaccasesalt - T3wheatstrawlaccasenosalt : -4.204013 (0.0025)*
T2wheatstrawlaccasenosalt - T3wheatstrawlaccasenosalt : -0.066730 (1.0000)
T2wheatstrawlaccasesalt - T3wheatstrawlaccasenosalt : -0.525501 (1.0000)
T3seagrasslaccasenosalt - T3wheatstrawlaccasenosalt : -2.335562 (1.0000)
T3seagrasslaccasesalt - T3wheatstrawlaccasenosalt : -4.383350 (0.0011)*
ASPseagrasslaccasenosalt - T3wheatstrawlaccasesalt : -3.607610 (0.0294)
ASPseagrasslaccasesalt - T3wheatstrawlaccasesalt : -3.795289 (0.0140)*
ASPwheatstrawlaccasenosalt - T3wheatstrawlaccasesalt : -2.744286 (0.5761)
ASPwheatstrawlaccasesalt - T3wheatstrawlaccasesalt : -4.320791 (0.0015)*
STYseagrasslaccasenosalt - T3wheatstrawlaccasesalt : -2.035276 (1.0000)
STYseagrasslaccasesalt - T3wheatstrawlaccasesalt : -1.367972 (1.0000)
STYwheatstrawlaccasenosalt - T3wheatstrawlaccasesalt : -0.767399 (1.0000)
STYwheatstrawlaccasesalt - T3wheatstrawlaccasesalt : -2.293856 (1.0000)
T1seagrasslaccasenosalt - T3wheatstrawlaccasesalt : -1.234511 (1.0000)
T1seagrasslaccasesalt - T3wheatstrawlaccasesalt : -3.615951 (0.0284)
T1wheatstrawlaccasenosalt - T3wheatstrawlaccasesalt : 0.900859 (1.0000)
T1wheatstrawlaccasesalt - T3wheatstrawlaccasesalt : -0.183508 (1.0000)
T2seagrasslaccasenosalt - T3wheatstrawlaccasesalt : -1.739160 (1.0000)
T2seagrasslaccasesalt - T3wheatstrawlaccasesalt : -3.461637 (0.0510)
T2wheatstrawlaccasenosalt - T3wheatstrawlaccasesalt : 0.675644 (1.0000)
T2wheatstrawlaccasesalt - T3wheatstrawlaccasesalt : 0.216873 (1.0000)
T3seagrasslaccasenosalt - T3wheatstrawlaccasesalt : -1.593187 (1.0000)
T3seagrasslaccasesalt - T3wheatstrawlaccasesalt : -3.640975 (0.0258)
T3wheatstrawlaccasenosalt - T3wheatstrawlaccasesalt : 0.742375 (1.0000)

alpha = 0.05
Reject Ho if p <= alpha/2

GROUPING

cldList(P.adj ~ Comparison,data=PT, threshold+0,05)

	Group	Letter	MonoLetter
1	ASPseagrasslaccasenosalt	abc	abc
2	ASPseagrasslaccasesalt	ab	ab
3	ASPwheatstrawlaccasenosalt	abcde	abcde
4	ASPwheatstrawlaccasesalt	a	a

5 STYseagrasslaccasenosalt abcdefg abcdefg
6 STYseagrasslaccasesalt cdefghi cdefghi
7 STYwheatstrawlaccasenosalt dfghi d fghi
8 STYwheatstrawlaccasesalt abcdef abcdef
9 T1seagrasslaccasenosalt defghi defghi
10 T1seagrasslaccasesalt abc abc
11 T1wheatstrawlaccasenosalt h h
12 T1wheatstrawlaccasesalt fghi fghi
13 T2seagrasslaccasenosalt bcdefg bcdefg
14 T2seagrasslaccasesalt abce abc e
15 T2wheatstrawlaccasenosalt hi hi
16 T2wheatstrawlaccasesalt ghi ghi
17 T3seagrasslaccasenosalt bcdefgi bcdefg i
18 T3seagrasslaccasesalt abc abc
19 T3wheatstrawlaccasenosalt hi hi
20 T3wheatstrawlaccasesalt fghi fghi

2) ACTIVITY CELLULASE

No significant differences were found between the differents groups.

3) ACTIVITY XYLANASE

Kruskal-Wallis rank sum test :

Kruskal-Wallis chi-squared = 76.256, df = 19, p-value = 8.146e-09

RESULTS OF THE DUNN PAIRWISE MULTIPLE TEST COMPARISON

Dunn (1964) Kruskal-Wallis multiple comparison p-values adjusted with the Benjamini-Hochberg method

	Comparison	Z	P.unadj	P.adj
1	ASPseagrassxylanenosalt - ASPseagrassxylanesalt	-0.43158545	6.660427e-01	8.325534e-01
2	ASPseagrassxylanenosalt - ASPwheatstrawxylanenosalt	-2.09982919	3.574387e-02	1.257655e-01
3	ASPseagrassxylanesalt - ASPwheatstrawxylanenosalt	-1.66824374	9.526735e-02	2.033797e-01
4	ASPseagrassxylanenosalt - ASPwheatstrawxylanesalt	0.00000000	1.000000e+00	1.000000e+00
5	ASPseagrassxylanesalt - ASPwheatstrawxylanesalt	0.43158545	6.660427e-01	8.380670e-01
6	ASPwheatstrawxylanenosalt - ASPwheatstrawxylanesalt	2.09982919	3.574387e-02	1.281384e-01
7	ASPseagrassxylanenosalt - STYseagrassxylanenosalt	1.27815690	2.011941e-01	3.295421e-01
8	ASPseagrassxylanesalt - STYseagrassxylanenosalt	1.70974234	8.731353e-02	1.906847e-01
9	ASPwheatstrawxylanenosalt - STYseagrassxylanenosalt	3.37798609	7.301878e-04	8.160922e-03
10	ASPwheatstrawxylanesalt - STYseagrassxylanenosalt	1.27815690	2.011941e-01	3.324077e-01
11	ASPseagrassxylanenosalt - STYseagrassxylanesalt	-2.27412331	2.295857e-02	9.913929e-02
12	ASPseagrassxylanesalt - STYseagrassxylanesalt	-1.84253786	6.539651e-02	1.656712e-01
13	ASPwheatstrawxylanenosalt - STYseagrassxylanesalt	-0.17429412	8.616343e-01	9.630031e-01
14	ASPwheatstrawxylanesalt - STYseagrassxylanesalt	-2.27412331	2.295857e-02	1.014449e-01
15	STYseagrassxylanenosalt - STYseagrassxylanesalt	-3.55228021	3.819079e-04	5.581731e-03
16	ASPseagrassxylanenosalt - STYwheatstrawxylanenosalt	-0.87977033	3.789837e-01	5.538993e-01

17 ASPseagrassxylanesalt - STYwheatstrawxylanenosalt -0.44818489 6.540198e-01 8.284250e-01
18 ASPwheatstrawxylanenosalt - STYwheatstrawxylanenosalt 1.22005886 2.224426e-01 3.551604e-01
19 ASPwheatstrawxylanesalt - STYwheatstrawxylanenosalt -0.87977033 3.789837e-01 5.581931e-01
20 STYseagrassxylanenosalt - STYwheatstrawxylanenosalt -2.15792723 3.093349e-02 1.199462e-01
21 STYseagrassxylanesalt - STYwheatstrawxylanenosalt 1.39435298 1.632110e-01 3.010689e-01
22 ASPseagrassxylanenosalt - STYwheatstrawxylanesalt 1.27815690 2.011941e-01 3.353235e-01
23 ASPseagrassxylanesalt - STYwheatstrawxylanesalt 1.70974234 8.731353e-02 1.929020e-01
24 ASPwheatstrawxylanenosalt - STYwheatstrawxylanesalt 3.37798609 7.301878e-04 8.670980e-03
25 ASPwheatstrawxylanesalt - STYwheatstrawxylanesalt 1.27815690 2.011941e-01 3.382910e-01
26 STYseagrassxylanenosalt - STYwheatstrawxylanesalt 0.00000000 1.000000e+00 1.000000e+00
27 STYseagrassxylanesalt - STYwheatstrawxylanesalt 3.55228021 3.819079e-04 6.046876e-03
28 STYwheatstrawxylanenosalt - STYwheatstrawxylanesalt 2.15792723 3.093349e-02 1.224451e-01
29 ASPseagrassxylanenosalt - T1seagrassxylanenosalt -1.92553507 5.416245e-02 1.470124e-01
30 ASPseagrassxylanesalt - T1seagrassxylanenosalt -1.49394962 1.351888e-01 2.675611e-01
31 ASPwheatstrawxylanenosalt - T1seagrassxylanenosalt 0.17429412 8.616343e-01 9.687013e-01
32 ASPwheatstrawxylanesalt - T1seagrassxylanenosalt -1.92553507 5.416245e-02 1.491430e-01
33 STYseagrassxylanenosalt - T1seagrassxylanenosalt -3.20369196 1.356776e-03 8.889219e-03
34 STYseagrassxylanesalt - T1seagrassxylanenosalt 0.34858824 7.273985e-01 8.859340e-01
35 STYwheatstrawxylanenosalt - T1seagrassxylanenosalt -1.04576473 2.956697e-01 4.494179e-01
36 STYwheatstrawxylanesalt - T1seagrassxylanenosalt -3.20369196 1.356776e-03 9.206691e-03
37 ASPseagrassxylanenosalt - T1seagrassxylanesalt -2.61441184 8.938123e-03 4.717342e-02
38 ASPseagrassxylanesalt - T1seagrassxylanesalt -2.18282639 2.904860e-02 1.226496e-01
39 ASPwheatstrawxylanenosalt - T1seagrassxylanesalt -0.51458265 6.068447e-01 7.790574e-01
40 ASPwheatstrawxylanesalt - T1seagrassxylanesalt -2.61441184 8.938123e-03 4.852124e-02
41 STYseagrassxylanenosalt - T1seagrassxylanesalt -3.89256873 9.918839e-05 1.884579e-03
42 STYseagrassxylanesalt - T1seagrassxylanesalt -0.34028852 7.336393e-01 8.878437e-01
43 STYwheatstrawxylanenosalt - T1seagrassxylanesalt -1.73464150 8.280432e-02 1.895521e-01
44 STYwheatstrawxylanesalt - T1seagrassxylanesalt -3.89256873 9.918839e-05 2.093977e-03
45 T1seagrassxylanenosalt - T1seagrassxylanesalt -0.68887677 4.909008e-01 6.808114e-01
46 ASPseagrassxylanenosalt - T1wheatstrawxylanenosalt -3.40288525 6.667828e-04 8.445915e-03
47 ASPseagrassxylanesalt - T1wheatstrawxylanenosalt -2.97129980 2.965421e-03 1.707364e-02
48 ASPwheatstrawxylanenosalt - T1wheatstrawxylanenosalt -1.30305606 1.925556e-01 3.325961e-01
49 ASPwheatstrawxylanesalt - T1wheatstrawxylanenosalt -3.40288525 6.667828e-04 9.049195e-03
50 STYseagrassxylanenosalt - T1wheatstrawxylanenosalt -4.68104214 2.854203e-06 1.355746e-04
51 STYseagrassxylanesalt - T1wheatstrawxylanenosalt -1.12876194 2.589983e-01 4.100806e-01
52 STYwheatstrawxylanenosalt - T1wheatstrawxylanenosalt -2.52311491 1.163204e-02 5.666890e-02
53 STYwheatstrawxylanesalt - T1wheatstrawxylanenosalt -4.68104214 2.854203e-06 1.807662e-04
54 T1seagrassxylanenosalt - T1wheatstrawxylanenosalt -1.47735018 1.395818e-01 2.706178e-01
55 T1seagrassxylanesalt - T1wheatstrawxylanenosalt -0.78847341 4.304198e-01 6.195437e-01
56 ASPseagrassxylanenosalt - T1wheatstrawxylanesalt -1.92553507 5.416245e-02 1.513363e-01
57 ASPseagrassxylanesalt - T1wheatstrawxylanesalt -1.49394962 1.351888e-01 2.703776e-01
58 ASPwheatstrawxylanenosalt - T1wheatstrawxylanesalt 0.17429412 8.616343e-01 9.744674e-01
59 ASPwheatstrawxylanesalt - T1wheatstrawxylanesalt -1.92553507 5.416245e-02 1.535950e-01
60 STYseagrassxylanenosalt - T1wheatstrawxylanesalt -3.20369196 1.356776e-03 9.547680e-03
61 STYseagrassxylanesalt - T1wheatstrawxylanesalt 0.34858824 7.273985e-01 8.916497e-01
62 STYwheatstrawxylanenosalt - T1wheatstrawxylanesalt -1.04576473 2.956697e-01 4.530422e-01
63 STYwheatstrawxylanesalt - T1wheatstrawxylanesalt -3.20369196 1.356776e-03 9.914898e-03
64 T1seagrassxylanenosalt - T1wheatstrawxylanesalt 0.00000000 1.000000e+00 1.000000e+00
65 T1seagrassxylanesalt - T1wheatstrawxylanesalt 0.68887677 4.909008e-01 6.858173e-01

66	T1wheatstrawxylanenosalt - T1wheatstrawxylanesalt	1.47735018	1.395818e-01	2.734076e-01
67	ASPseagrassxylanenosalt - T2seagrassxylanenosalt	-1.97533339	4.823033e-02	1.454565e-01
68	ASPseagrassxylanesalt - T2seagrassxylanenosalt	-1.54374794	1.226494e-01	2.505741e-01
69	ASPwheatstrawxylanenosalt - T2seagrassxylanenosalt	0.12449580	9.009227e-01	9.894527e-01
70	ASPwheatstrawxylanesalt - T2seagrassxylanenosalt	-1.97533339	4.823033e-02	1.478026e-01
71	STYseagrassxylanenosalt - T2seagrassxylanenosalt	-3.25349028	1.139966e-03	9.417112e-03
72	STYseagrassxylanesalt - T2seagrassxylanenosalt	0.29878992	7.651003e-01	8.973399e-01
73	STYwheatstrawxylanenosalt - T2seagrassxylanenosalt	-1.09556305	2.732700e-01	4.255845e-01
74	STYwheatstrawxylanesalt - T2seagrassxylanenosalt	-3.25349028	1.139966e-03	9.845163e-03
75	T1seagrassxylanenosalt - T2seagrassxylanenosalt	-0.04979832	9.602831e-01	1.000000e+00
76	T1seagrassxylanesalt - T2seagrassxylanenosalt	0.63907845	5.227719e-01	6.945920e-01
77	T1wheatstrawxylanenosalt - T2seagrassxylanenosalt	1.42755186	1.534209e-01	2.857840e-01
78	T1wheatstrawxylanesalt - T2seagrassxylanenosalt	-0.04979832	9.602831e-01	1.000000e+00
79	ASPseagrassxylanenosalt - T2seagrassxylanesalt	-1.32795522	1.841929e-01	3.210702e-01
80	ASPseagrassxylanesalt - T2seagrassxylanesalt	-0.89636977	3.700553e-01	5.536260e-01
81	ASPwheatstrawxylanenosalt - T2seagrassxylanesalt	0.77187397	4.401891e-01	6.195254e-01
82	ASPwheatstrawxylanesalt - T2seagrassxylanesalt	-1.32795522	1.841929e-01	3.240431e-01
83	STYseagrassxylanenosalt - T2seagrassxylanesalt	-2.60611212	9.157648e-03	4.578824e-02
84	STYseagrassxylanesalt - T2seagrassxylanesalt	0.94616809	3.440629e-01	5.188249e-01
85	STYwheatstrawxylanenosalt - T2seagrassxylanesalt	-0.44818489	6.540198e-01	8.339849e-01
86	STYwheatstrawxylanesalt - T2seagrassxylanesalt	-2.60611212	9.157648e-03	4.702576e-02
87	T1seagrassxylanenosalt - T2seagrassxylanesalt	0.59757985	5.501203e-01	7.159100e-01
88	T1seagrassxylanesalt - T2seagrassxylanesalt	1.28645662	1.982837e-01	3.363742e-01
89	T1wheatstrawxylanenosalt - T2seagrassxylanesalt	2.07493003	3.799302e-02	1.266434e-01
90	T1wheatstrawxylanesalt - T2seagrassxylanesalt	0.59757985	5.501203e-01	7.208473e-01
91	T2seagrassxylanenosalt - T2seagrassxylanesalt	0.64737817	5.173872e-01	6.971885e-01
92	ASPseagrassxylanenosalt - T2wheatstrawxylanenosalt	-4.06686285	4.765027e-05	1.508925e-03
93	ASPseagrassxylanesalt - T2wheatstrawxylanenosalt	-3.63527741	2.776817e-04	4.796320e-03
94	ASPwheatstrawxylanenosalt - T2wheatstrawxylanenosalt	-1.96703367	4.917933e-02	1.460011e-01
95	ASPwheatstrawxylanesalt - T2wheatstrawxylanenosalt	-4.06686285	4.765027e-05	1.810710e-03
96	STYseagrassxylanenosalt - T2wheatstrawxylanenosalt	-5.34501975	9.040719e-08	8.588683e-06
97	STYseagrassxylanesalt - T2wheatstrawxylanenosalt	-1.79273954	7.301458e-02	1.691801e-01
98	STYwheatstrawxylanenosalt - T2wheatstrawxylanenosalt	-3.18709252	1.437108e-03	9.101684e-03
99	STYwheatstrawxylanesalt - T2wheatstrawxylanenosalt	-5.34501975	9.040719e-08	1.717737e-05
100	T1seagrassxylanenosalt - T2wheatstrawxylanenosalt	-2.14132779	3.224761e-02	1.201382e-01
101	T1seagrassxylanesalt - T2wheatstrawxylanenosalt	-1.45245102	1.463762e-01	2.781149e-01
102	T1wheatstrawxylanenosalt - T2wheatstrawxylanenosalt	-0.66397761	5.067046e-01	6.926179e-01
103	T1wheatstrawxylanesalt - T2wheatstrawxylanenosalt	-2.14132779	3.224761e-02	1.225409e-01
104	T2seagrassxylanenosalt - T2wheatstrawxylanenosalt	-2.09152947	3.648063e-02	1.237736e-01
105	T2seagrassxylanesalt - T2wheatstrawxylanenosalt	-2.73890764	6.164369e-03	3.444794e-02
106	ASPseagrassxylanenosalt - T2wheatstrawxylanesalt	-1.97533339	4.823033e-02	1.502256e-01
107	ASPseagrassxylanesalt - T2wheatstrawxylanesalt	-1.54374794	1.226494e-01	2.532977e-01
108	ASPwheatstrawxylanenosalt - T2wheatstrawxylanesalt	0.12449580	9.009227e-01	9.952053e-01
109	ASPwheatstrawxylanesalt - T2wheatstrawxylanesalt	-1.97533339	4.823033e-02	1.527294e-01
110	STYseagrassxylanenosalt - T2wheatstrawxylanesalt	-3.25349028	1.139966e-03	1.031398e-02
111	STYseagrassxylanesalt - T2wheatstrawxylanesalt	0.29878992	7.651003e-01	9.029134e-01
112	STYwheatstrawxylanenosalt - T2wheatstrawxylanesalt	-1.09556305	2.732700e-01	4.291017e-01
113	STYwheatstrawxylanesalt - T2wheatstrawxylanesalt	-3.25349028	1.139966e-03	1.082968e-02
114	T1seagrassxylanenosalt - T2wheatstrawxylanesalt	-0.04979832	9.602831e-01	1.000000e+00

115 T1seagrassxylanesalt - T2wheatstrawxylanesalt 0.63907845 5.227719e-01 6.994835e-01

116 T1wheatstrawxylanenosalt - T2wheatstrawxylanesalt 1.42755186 1.534209e-01 2.886136e-01

117 T1wheatstrawxylanesalt - T2wheatstrawxylanesalt -0.04979832 9.602831e-01 1.000000e+00

118 T2seagrassxylanenosalt - T2wheatstrawxylanesalt 0.00000000 1.000000e+00 1.000000e+00

119 T2seagrassxylanesalt - T2wheatstrawxylanesalt -0.64737817 5.173872e-01 7.021684e-01

120 T2wheatstrawxylanenosalt - T2wheatstrawxylanesalt 2.09152947 3.648063e-02 1.260240e-01

121 ASPseagrassxylanenosalt - T3seagrassxylanenosalt -0.10789636 9.140779e-01 9.812136e-01

122 ASPseagrassxylanesalt - T3seagrassxylanenosalt 0.32368908 7.461734e-01 8.860810e-01

123 ASPwheatstrawxylanenosalt - T3seagrassxylanenosalt 1.99193283 4.637843e-02 1.493543e-01

124 ASPwheatstrawxylanesalt - T3seagrassxylanenosalt -0.10789636 9.140779e-01 9.867886e-01

125 STYseagrassxylanenosalt - T3seagrassxylanenosalt -1.38605326 1.657306e-01 2.942881e-01

126 STYseagrassxylanesalt - T3seagrassxylanenosalt 2.16622695 3.029385e-02 1.224645e-01

127 STYwheatstrawxylanenosalt - T3seagrassxylanenosalt 0.77187397 4.401891e-01 6.241487e-01

128 STYwheatstrawxylanesalt - T3seagrassxylanenosalt -1.38605326 1.657306e-01 2.970644e-01

129 T1seagrassxylanenosalt - T3seagrassxylanenosalt 1.81763870 6.911937e-02 1.621319e-01

130 T1seagrassxylanesalt - T3seagrassxylanenosalt 2.50651547 1.219277e-02 5.650309e-02

131 T1wheatstrawxylanenosalt - T3seagrassxylanenosalt 3.29498888 9.842557e-04 9.842557e-03

132 T1wheatstrawxylanesalt - T3seagrassxylanenosalt 1.81763870 6.911937e-02 1.641585e-01

133 T2seagrassxylanenosalt - T3seagrassxylanenosalt 1.86743703 6.184058e-02 1.587799e-01

134 T2seagrassxylanesalt - T3seagrassxylanenosalt 1.22005886 2.224426e-01 3.581702e-01

135 T2wheatstrawxylanenosalt - T3seagrassxylanenosalt 3.95896649 7.527480e-05 1.787776e-03

136 T2wheatstrawxylanesalt - T3seagrassxylanenosalt 1.86743703 6.184058e-02 1.609549e-01

137 ASPseagrassxylanenosalt - T3seagrassxylanesalt -1.72634178 8.428594e-02 1.884039e-01

138 ASPseagrassxylanesalt - T3seagrassxylanesalt -1.29475634 1.954043e-01 3.344758e-01

139 ASPwheatstrawxylanenosalt - T3seagrassxylanesalt 0.37348741 7.087857e-01 8.744759e-01

140 ASPwheatstrawxylanesalt - T3seagrassxylanesalt -1.72634178 8.428594e-02 1.906468e-01

141 STYseagrassxylanenosalt - T3seagrassxylanesalt -3.00449868 2.660189e-03 1.579487e-02

142 STYseagrassxylanesalt - T3seagrassxylanesalt 0.54778153 5.838419e-01 7.546256e-01

143 STYwheatstrawxylanenosalt - T3seagrassxylanesalt -0.84657145 3.972340e-01 5.761410e-01

144 STYwheatstrawxylanesalt - T3seagrassxylanesalt -3.00449868 2.660189e-03 1.630438e-02

145 T1seagrassxylanenosalt - T3seagrassxylanesalt 0.19919328 8.421116e-01 9.580910e-01

146 T1seagrassxylanesalt - T3seagrassxylanesalt 0.88807005 3.745031e-01 5.559030e-01

147 T1wheatstrawxylanenosalt - T3seagrassxylanesalt 1.67654346 9.363179e-02 2.021595e-01

148 T1wheatstrawxylanesalt - T3seagrassxylanesalt 0.19919328 8.421116e-01 9.638626e-01

149 T2seagrassxylanenosalt - T3seagrassxylanesalt 0.24899160 8.033673e-01 9.307304e-01

150 T2seagrassxylanesalt - T3seagrassxylanesalt -0.39838657 6.903453e-01 8.572915e-01

151 T2wheatstrawxylanenosalt - T3seagrassxylanesalt 2.34052107 1.925685e-02 8.711433e-02

152 T2wheatstrawxylanesalt - T3seagrassxylanesalt 0.24899160 8.033673e-01 9.364404e-01

153 T3seagrassxylanenosalt - T3seagrassxylanesalt -1.61844542 1.055666e-01 2.204139e-01

154 ASPseagrassxylanenosalt - T3wheatstrawxylanenosalt -1.94213451 5.212082e-02 1.500448e-01

155 ASPseagrassxylanesalt - T3wheatstrawxylanenosalt -1.51054906 1.309034e-01 2.645919e-01

156 ASPwheatstrawxylanenosalt - T3wheatstrawxylanenosalt 0.15769468 8.746974e-01 9.718860e-01

157 ASPwheatstrawxylanesalt - T3wheatstrawxylanenosalt -1.94213451 5.212082e-02 1.523532e-01

158 STYseagrassxylanenosalt - T3wheatstrawxylanenosalt -3.22029140 1.280604e-03 9.732587e-03

159 STYseagrassxylanesalt - T3wheatstrawxylanenosalt 0.33198880 7.398977e-01 8.897504e-01

160 STYwheatstrawxylanenosalt - T3wheatstrawxylanenosalt -1.06236417 2.880704e-01 4.449868e-01

161 STYwheatstrawxylanesalt - T3wheatstrawxylanenosalt -3.22029140 1.280604e-03 1.013811e-02

162 T1seagrassxylanenosalt - T3wheatstrawxylanenosalt -0.01659944 9.867562e-01 1.000000e+00

163 T1seagrassxylanesalt - T3wheatstrawxylanenosalt 0.67227733 5.014072e-01 6.903432e-01

164	T1wheatstrawxylanenosalt - T3wheatstrawxylanenosalt	1.46075074	1.440839e-01	2.765246e-01
165	T1wheatstrawxylanesalt - T3wheatstrawxylanenosalt	-0.01659944	9.867562e-01	1.000000e+00
166	T2seagrassxylanenosalt - T3wheatstrawxylanenosalt	0.03319888	9.735160e-01	1.000000e+00
167	T2seagrassxylanesalt - T3wheatstrawxylanenosalt	-0.61417929	5.390969e-01	7.113084e-01
168	T2wheatstrawxylanenosalt - T3wheatstrawxylanenosalt	2.12472835	3.360929e-02	1.228032e-01
169	T2wheatstrawxylanesalt - T3wheatstrawxylanenosalt	0.03319888	9.735160e-01	1.000000e+00
170	T3seagrassxylanenosalt - T3wheatstrawxylanenosalt	-1.83423814	6.661865e-02	1.643837e-01
171	T3seagrassxylanesalt - T3wheatstrawxylanenosalt	-0.21579272	8.291493e-01	9.547780e-01
172	ASPseagrassxylanenosalt - T3wheatstrawxylanesalt	-0.10789636	9.140779e-01	9.924274e-01
173	ASPseagrassxylanesalt - T3wheatstrawxylanesalt	0.32368908	7.461734e-01	8.916538e-01
174	ASPwheatstrawxylanenosalt - T3wheatstrawxylanesalt	1.99193283	4.637843e-02	1.519293e-01
175	ASPwheatstrawxylanesalt - T3wheatstrawxylanesalt	-0.10789636	9.140779e-01	9.981310e-01
176	STYseagrassxylanenosalt - T3wheatstrawxylanesalt	-1.38605326	1.657306e-01	2.998935e-01
177	STYseagrassxylanesalt - T3wheatstrawxylanesalt	2.16622695	3.029385e-02	1.251268e-01
178	STYwheatstrawxylanenosalt - T3wheatstrawxylanesalt	0.77187397	4.401891e-01	6.288415e-01
179	STYwheatstrawxylanesalt - T3wheatstrawxylanesalt	-1.38605326	1.657306e-01	3.027771e-01
180	T1seagrassxylanenosalt - T3wheatstrawxylanesalt	1.81763870	6.911937e-02	1.662365e-01
181	T1seagrassxylanesalt - T3wheatstrawxylanesalt	2.50651547	1.219277e-02	5.791567e-02
182	T1wheatstrawxylanenosalt - T3wheatstrawxylanesalt	3.29498888	9.842557e-04	1.038937e-02
183	T1wheatstrawxylanesalt - T3wheatstrawxylanesalt	1.81763870	6.911937e-02	1.683677e-01
184	T2seagrassxylanenosalt - T3wheatstrawxylanesalt	1.86743703	6.184058e-02	1.631904e-01
185	T2seagrassxylanesalt - T3wheatstrawxylanesalt	1.22005886	2.224426e-01	3.612315e-01
186	T2wheatstrawxylanenosalt - T3wheatstrawxylanesalt	3.95896649	7.527480e-05	2.043173e-03
187	T2wheatstrawxylanesalt - T3wheatstrawxylanesalt	1.86743703	6.184058e-02	1.654889e-01
188	T3seagrassxylanenosalt - T3wheatstrawxylanesalt	0.00000000	1.000000e+00	1.000000e+00
189	T3seagrassxylanesalt - T3wheatstrawxylanesalt	1.61844542	1.055666e-01	2.228629e-01
190	T3wheatstrawxylanenosalt - T3wheatstrawxylanesalt	1.83423814	6.661865e-02	1.665466e-01

GROUPING

	Group	Letter	MonoLetter
1	ASPseagrassxylanenosalt	ab	ab
2	ASPseagrassxylanesalt	abc	abc
3	ASPwheatstrawxylanenosalt	bcde	bcde
4	ASPwheatstrawxylanesalt	ab	ab
5	STYseagrassxylanenosalt	e	e
6	STYseagrassxylanesalt	bcde	bcde
7	STYwheatstrawxylanenosalt	abcd	abcd
8	STYwheatstrawxylanesalt	a	a
9	T1seagrassxylanenosalt	bcde	bcde
10	T1seagrassxylanesalt	cde	cde
11	T1wheatstrawxylanenosalt	de	de
12	T1wheatstrawxylanesalt	bcde	bcde
13	T2seagrassxylanenosalt	bcde	bcde
14	T2seagrassxylanesalt	bcd	bcd
15	T2wheatstrawxylanenosalt	e	e
16	T2wheatstrawxylanesalt	bcde	bcde
17	T3seagrassxylanenosalt	abc	abc
18	T3seagrassxylanesalt	bcde	bcde

19 T3wheatstrawxylanenosalt bcde bcde
20 T3wheatstrawxylanesalt abc abc
>
